# 3D-printed hyaluronic acid hydrogel scaffolds impregnated with neurotrophic factors (BDNF, GDNF) for post-traumatic brain tissue reconstruction

**DOI:** 10.3389/fbioe.2022.895406

**Published:** 2022-08-25

**Authors:** Tatiana A. Mishchenko, Maria O. Klimenko, Alisa I. Kuznetsova, Roman S. Yarkov, Alexander G. Savelyev, Anastasia V. Sochilina, Alexandra O. Mariyanats, Vladimir K. Popov, Evgeny V. Khaydukov, Andrei V. Zvyagin, Maria V. Vedunova

**Affiliations:** ^1^ Institute of Biology and Biomedicine, National Research Lobachevsky State University of Nizhny Novgorod, Nizhny Novgorod, Russia; ^2^ Federal Scientific Research Centre “Crystallography and Photonics”, Russian Academy of Sciences, Troitsk-Moscow, Russia; ^3^ Sechenov First Moscow State Medical University, Moscow, Russia; ^4^ Shemyakin-Ovchinnikov Institute of Bioorganic Chemistry RAS, Moscow, Russia; ^5^ MQ Photonics Centre, Macquarie University, Sydney, NSW, Australia

**Keywords:** brain trauma, 3D printing, scaffold, neurotrophic factors BDNF and GDNF, neurotransplantation, biocompatibility

## Abstract

Brain tissue reconstruction posttraumatic injury remains a long-standing challenge in neurotransplantology, where a tissue-engineering construct (scaffold, SC) with specific biochemical properties is deemed the most essential building block. Such three-dimensional (3D) hydrogel scaffolds can be formed using brain-abundant endogenous hyaluronic acid modified with glycidyl methacrylate by employing our proprietary photopolymerisation technique. Herein, we produced 3D hyaluronic scaffolds impregnated with neurotrophic factors (BDNF, GDNF) possessing 600 kPa Young’s moduli and 336% swelling ratios. Stringent *in vitro* testing of fabricated scaffolds using primary hippocampal cultures revealed lack of significant cytotoxicity: the number of viable cells in the SC+BDNF (91.67 ± 1.08%) and SC+GDNF (88.69 ± 1.2%) groups was comparable to the sham values (*p* > 0.05). Interestingly, BDNF-loaded scaffolds promoted the stimulation of neuronal process outgrowth during the first 3 days of cultures development (day 1: 23.34 ± 1.46 µm; day 3: 37.26 ± 1.98 µm, *p* < 0.05, vs*.* sham), whereas GDNF-loaded scaffolds increased the functional activity of neuron-glial networks of cultures at later stages of cultivation (day 14) manifested in a 1.3-fold decrease in the duration coupled with a 2.4-fold increase in the frequency of Ca^2+^ oscillations (*p* < 0.05, vs*.* sham). *In vivo* studies were carried out using C57BL/6 mice with induced traumatic brain injury, followed by surgery augmented with scaffold implantation. We found positive dynamics of the morphological changes in the treated nerve tissue in the post-traumatic period, where the GDNF-loaded scaffolds indicated more favorable regenerative potential. In comparison with controls, the physiological state of the treated mice was improved manifested by the absence of severe neurological deficit, significant changes in motor and orienting-exploratory activity, and preservation of the ability to learn and retain long-term memory. Our results suggest in favor of biocompatibility of GDNF-loaded scaffolds, which provide a platform for personalized brain implants stimulating effective morphological and functional recovery of nerve tissue after traumatic brain injury.

## Introduction

The problem of morphological and functional recovery of nerve tissue after traumatic brain injury (TBI) remains one of the most challenging areas in neurobiological and medical sciences. The severe condition of the patient is mediated not only by mechanical cell death occurring at the time of injury but also by the launch of several pathological processes leading to the loss of functional activity of neural networks and consequently to the development of acute neurological deficits, epilepsy, mental illnesses, impaired mnestic and cognitive functions and, ultimately, profound disability (Kaur and Sharma, 2018; Killen et al., 2019). The low therapeutic efficacy of current strategies dictates the need to develop new approaches that prevent the development of pathological reactions and stimulate endogenous processes of functional regeneration.

Over the past decade, the research interest in the design of personalized bioengineered constructs, i.e., scaffolds, for brain tissue reconstruction after injuries of various origin has been emerged. Scaffolds (SC) help to maintain the brain’s anatomical structure in the damaged area, supporting cellular spatial distribution, free transport of biological fluids and vascularization, with the possibility of gradual replacement of the transplant with natural nerve tissue during its bioresorption (Mahumane et al., 2018). Despite the significant relevance of the use of SC in neurotransplantology, the design and fabrication of three-dimensional (3D) constructs still faced with several challenges compromising their clinical application. The primary debatable issue is the selection of scaffold material, which must be highly biocompatible with nerve cells and minimally cytotoxic during its biodegradation (Mahumane et al., 2018; Smith et al., 2018; Tan et al., 2020).

Hyaluronic acid (HA) is considered the most promising material for creating neurotransplants. As it is the main structural component of the brain’s extracellular matrix, HA-based scaffolds could be highly adherent and also biocompatible with nervous system cells (Neuman et al., 2015; Jensen et al., 2020). HA is also an antioxidant that contributes to the neutralization of the large quantities of free radicals produced in the brain during trauma; it is also involved in cell differentiation, neuronal proliferation, cell migration, morphogenesis, angiogenesis (Sudha and Rose, 2014) and synaptic plasticity (Kochlamazashvili et al., 2010; Rusakov and Dityatev, 2014). HA can be modified by the conjugation of chemical reagents containing double bonds with the polysaccharide backbone. The modified HA can be photocured, which makes it a promising, technologically advanced material for 3D printing applications (Savelyev et al., 2018; Später et al., 2020). Recent studies have been demonstrated photocrosslinkable hydrogels based on glycidyl methacrylate-modified HA whose tunable mechanical properties are capable of recapitulating the viscoelastic nature of the extracellular matrix that provides an ability to develop scaffolds for a wide variety of soft tissue engineering applications, in particular for the nerve tissue regeneration (Spearman et al., 2020). Moreover, HA has good compatibility with other materials, and modern bioengineering methods can be used to modify its mechanical properties and achieve a certain biodegradation rate (Lu et al., 2019; Jensen et al., 2020). In recent experimental studies *in vivo*, HA-based scaffolds positively affected on tissue regeneration, cognitive functions, and long-term memory formation after brain injury that opens tempting future perspectives of their use for patients (Ghuman et al., 2018; Zhang et al., 2018; Lu et al., 2019).

Besides maintaining the natural anatomical structure in the damaged area, supplementation of scaffold material with biologically active substances could stimulate endogenous processes that ensure both cell survival and boosted restoration of the morpho-functional organization of neuron-glial networks. The neurotrophic factors BDNF and GDNF should be considered as potential stimulatory agents for reparative processes. These proteins perform a wide range of CNS functions: they are the regulators of synaptic plasticity and neurogenesis, and they participate directly in nerve cell adaptation and survival in several pathologies, including ischemia and neurodegenerative processes (Zuccato and Cattaneo, 2009; Xiao and Le, 2016; Castrén and Antila, 2017; Skaper, 2018; Walker and Xu, 2018; Mitroshina Е et al., 2019; Mitroshina et al., 2020). The positive neuroprotective and reparative effects of using SC loaded with neurotrophic factors have been shown in experimental transplantation in spinal cord injuries (Liu et al., 2018; Tom et al., 2018). Recent studies also demonstrate the possibility of using HA-based hydrogels impregnated with neurotrophic factors as a carrier for transplanted neural cells which have a great potential to improve their survival and proliferation in therapy of central nervous injuries (Nakaji-Hirabayashi et al., 2009; Wang et al., 2011). Moreover, a local depot release of neurotrophic factors from HA-based scaffold allows to provide the positive dynamics in experimental brain tissue recovery after stroke (Moshayedi et al., 2016; Cook et al., 2017). Thus, the application of HA-based hydrogels scaffolds loaded with neurotrophic factor (BDNF, GDNF) can provide a new avenue for personalized morpho-functional recovery of nerve tissue and improve therapeutic efficacy *via* negation the posttraumatic pathological consequences along with stimulation of functional regeneration of neuron-glial networks in the brain.

Herein, using the extrusion 3D printing technique, we designed and fabricated original scaffolds potentially intended for neurotransplantation. Our approach allows to conjugate natural (HA) and synthetic (glycidyl methacrylate (HAGM)) polymers and whereby achieve the controlled Young’s modulus and swelling ratios. The obtained mechanical properties of SC were close to those of brain tissue, as well as provide an opportunity to facilitate the loading of soluble neurotrophic factors (BDNF, GDNF) in water. Next, we analyzed the cytotoxic properties of SC loaded with BDNF, GDNF *in vitro*, and assessed their biocompatibility with nerve cells in the traumatic brain injury (TBI) model *in vivo*.

## Experimental

### Materials

The following materials were purchased from Sigma-Aldrich (United States): sodium hyaluronate (Mn = 100 kDa), glycidyl methacrylate (GMA), tetraethylammonium bromide, poly(ethylene glycol) diacrylate (PEG-DA, Mn = 575), acetone, N,N-dimethylformamide (DMF), and potassium permanganate (KMnO4). Flavin mononucleotide (FMN) was obtained from Pharmstandard (Russia) and triethanolamine (TEOHA) from Merck (United States). Phosphate buffered saline (PBS, pH 7.4) was prepared by dissolving a biotechnology grade PBS tablet (VWR Life Science, Canada) in 100 ml of deionized water. Penicillin-streptomycin (5000 U/ml and 5000 μg/ml respectively) was purchased from PanEco (Russia). Amphotericin B (5000 μg/ml) was purchased from JSC “Sintez” (Russia).

### Scaffolding

#### Modification of hyaluronic acid with glycidyl methacrylate

Modification of hyaluronic acid with glycidyl methacrylate was carried out according to the method described in (Sochilina et al., 2019). First, 1 g of sodium hyaluronate (salt form of HA) and 1 g of tetraethylammonium bromide were dissolved in 200 ml of deionized water, and then 66 ml of N,N-dimethylformamide and 14 ml of GMA were added to start the reaction. One milliliter of penicillin-streptomycin (5000 U/ml and 5000 μg/ml) and 250 µl of amphotericin B (5000 μg/ml) were added to prevent the possible growth of microorganisms. The reaction proceeded for 6 days with continuous stirring at 25°C. The resulting product, hyaluronic acid modified with glycidyl methacrylate (HAGM), was isolated by precipitation in seven-fold excess of acetone. HAGM was purified by dialysis against distilled water and then lyophilized in a FreeZone Freeze Dryer (Labconco, United States). The degree of substitution (DS) of HA disaccharide units with GMA was measured according to the colorimetric reaction protocol from (Sochilina et al., 2019). Briefly, the amount of GMA unsaturated units was determined by the reaction of HAGM solutions with potassium permanganate. DS was calculated as the percent ratio of the molar concentration of conjugated GMA groups to the general molar concentration of disaccharide units contained in the analyzed sample.

#### Preparation of photocurable composition

To produce a photocurable composition (PCC) capable of forming, by photocross-linking, entire cross-linked hydrogel volume with elastic properties, HAGM was dissolved in PCC at a concentration above the percolation threshold concentration, as discussed for 3D polymer networks in (Savelyev et al., 2017). Thus, HAGM, DS = 43% (17.5 wt%) and PEG-DA (5 wt%) were dissolved in the dark in deionized water. FMN (0.0014 wt%) with TEOHA (0.8 wt%) was added as a photoinitiating complex. The PCC was homogenized by sonication.

#### 3D printing

3D hydrogel structures were fabricated by extrusion printing as described in (Später et al., 2020). A removable glass syringe with a 150-μm nozzle was filled with PCC and the PCC was extruded onto the substrate by a motorized plunger. Patterning in X-Y directions was produced by moving the printing platform by two high-precision stepper motors. 3D structure printing was formed by using a layer-by-layer procedure according to the designed computer model. Two semiconductor CW, each with an intensity of up to 500 mW/cm^2^ at λ = 445 nm, were focused on the area around the nozzle. Irradiation activates the gelation process caused by PCC photocross-linking. The deposition of hydrogel fiber on the substrate was visually controlled by a CCD camera (Scopetek DCM130, China) equipped with a neutral-density light filter. Post-exposure at 30 mW/cm^2^ was conducted for 40 min to achieve homogeneous photocross-linking of hydrogel layers.

#### Hydrogel characterization

A mechanical testing machine (EZ-Test EZ-SX, Shimadzu, Japan) with a 500-N load cell was used for compressive tests to investigate the mechanical properties of the samples. Cylindrical samples with 5-mm diameter and 1-mm thickness were prepared by the micromolding technique and tested at the rate of 0.5 mm/min. Young’s moduli were automatically calculated according to the obtained stress-strain curves using the formula:

$$E=\frac{F/A_{0}}{\Delta L/L_{0}}$$ (1)

where F is a force applied to the sample, *L*_0_ is the gauge length, and *A*_0_ is the cross-sectional area of the sample, ΔL is the deformation due to the application of compressive stress. Strain was set as 70% of the gauge length.

For testing samples after swelling, we utilized Bioscope Resolve atomic force microscope (Bruker, United States) with ScanAsyst Fluid Plus triangular cantilever with a length of 70 μm and width of 10 μm as described in our previous work (Später et al., 2020). The nanoindentation of scaffolds was performed in the force mapping mode at 10 Hz scanning speed and 10 × 10 μm^2^ map size with 16 points. The samples were examined in a PBS solution at 37°C after timely swelling to a constant size. Young’s modulus was calculated on the Hertzian model.

Percent swelling ratios (SWR) of samples were studied by microgravimetry. Samples were freeze-dried and weighed using analytical balance AF 224RCE (VibRa, Japan). Then, the samples were placed in PBS for 10 min. After removing liquid drops, the samples were weighed again, and SWR was calculated according to the following formula:

$$\mathrm{SWR}\left(\%\right)=\;\left[\frac{m_{s}-m_{d}}{m_{d}}\right]\times \mathrm{100},$$ (2)

where *m*_*s*_ and *m*_*d*_ are the masses of swollen and dried samples, respectively.

#### Neurotrophic factors loading

The scaffolds were sterilized in aqueous solutions of ethanol (74.1%) and isopropanol (10%) for 15 min, exposed to UV light (365 nm) at 160 mW/cm^2^ for 30 min, and dried under sterile conditions. The neurotrophic factors BDNF (rhBDNF, 248-BDB, R&D Systems, United States) and GDNF (rhGDNF, 212-GD, R&D Systems, United States) were dissolved in water for 1 h in the concentration 30 ng/μl. The scaffolds were placed in an aqueous growth factor solution for 15 min, and then excess liquid on the scaffold surface was carefully removed. The final concentration of neurotrophic factors was approximately 100 ng per scaffold. Taking into account the SWR of the hydrogel (336%) and measuring $m_{d}$ we calculated that every scaffold soaked an average of 3.3 mg of the solution ( $m_{s}-m_{d}$ ). Therefore, the final concentration of neurotrophic factors can be estimated as 100 ng per scaffold. We believe that growth factor solution can be easily released after placing the sample in the liquid medium *in vitro* or *in vivo* as easily as it was loaded.

### Ethics statement

C57BL/6 mouse embryos obtained on the 18th day of gestation were used to prepare primary hippocampal cultures. *In vivo* experiments were carried out on adult male C57BL/6 mice (6–8 weeks of age, 25–28 g). The animals were housed in the specific-pathogen-free animal house of Lobachevsky University. All experimental procedures were performed in accordance with Act708n (23 August 2010) of the Ministry of Health of the Russian Federation, which describes the rules for laboratory care and use of laboratory animals, and Council Directive 2010/63 EU of the European Parliament (22 September 2010) on the protection of animals used for scientific purposes. The procedures were also approved by the Bioethics Committee of National Research Lobachevsky State University of Nizhny Novgorod (protocol No44 from 16 October 2020). The mice were killed by cervical dislocation, the embryos were surgically removed, and the animals were then decapitated.

### Isolation of primary hippocampal cultures

Primary hippocampal cultures were prepared and cultivated according to the protocol described by (Vedunova et al., 2015). Briefly, the hippocampi were surgically isolated from the embryonic brain and dissected in PBS, followed by 20 min of enzymatic treatment with 0.25% trypsin-EDTA solution (Thermo Fisher, 25200056, United States). Next, the cells were carefully resuspended in culture medium (Neurobasal^™^ medium; Thermo Fisher, 21103049, United States) supplemented with 2% B27 (Thermo Fisher, 175040446, United States), 0.5 mM L-glutamine (Thermo Fisher, 25030024, United States) and 5% fetal bovine serum (PanEco, K055, Russia) and centrifuged at 1000 rpm for 3 min.

**FIGURE 1:**
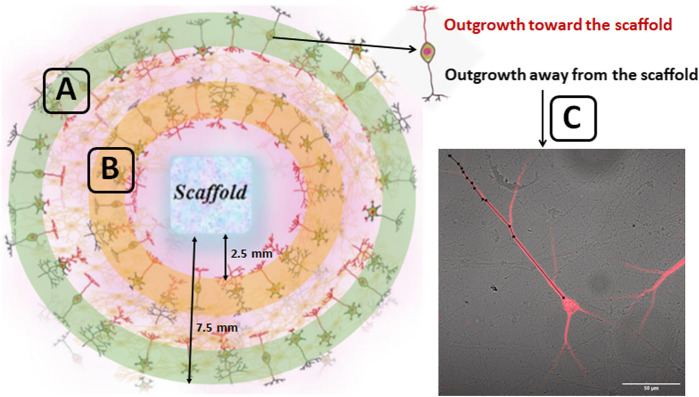
Schematic representation of counting regions of the outgrowth dynamics of neuronal processes in primary hippocampal culture. The cell suspension obtained from embryonic hippocampi was placed around the scaffold. The approximate distance of the inner and outer edge of the ring relative to the scaffold was 2.5 and 7.5 mm respectively. The outgrowth dynamics of neuronal processes was assessed in the 10 fields of view for distant (green zone) **(A)** as well as for adjustment areas (orange zone) **(B)** of cultures relative to the scaffold. The neuronal processes growing toward the scaffold (marked in red) and away from the scaffold (marked in black) were analyzed. The length of neuronal processes was counted from the neuron’s body to the terminus of the neurite **(C)** using ImageJ software.

The obtained cell suspension was placed around the scaffold (Figure 1), and after 20 min the culture medium was brought up to a standard volume. The initial density of cells was 4500 cells/mm^2^. The culture plates were pretreated with polyethyleneimine solution (1 mg/ml) (Sigma-Aldrich, P3143, Germany). The next day and every third day thereafter, 50% of the culture medium was refreshed with a medium containing a lower concentration of fetal bovine serum (0.4%). Cell viability was maintained under constant conditions of 35.5°C, 5% CO_2_ and a humidified atmosphere in a cell culture incubator for 21 days. The outgrowth dynamics of neuronal processes was analyzed on days 1, 3 and 7 of cultures development *in vitro* (DIV). To that end, images were obtained with an inverted microscope (Axio Observer A1; Zeiss, Germany) and analyzed in ImageJ software. The length of neuronal processes was measured from the neuron’s body to the terminus of the neurite in both the adjustment (10 fields of view) and distant areas (10 fields of view) of cultures relative to the scaffold (Figure 1). To confirm the performed analysis, the cultures underwent immunocytochemical validation.

### Immunocytochemical staining

The cellular content of primary hippocampal cultures was analyzed using immunocytochemical staining. The cultures were fixed with 4% paraformaldehyde solution supplemented with 4% sucrose for 15 min at room temperature and then incubated with a solution of 0.5% Triton X-100, 0.1% Tween 20, and 5% goat serum in PBS. Next, the cultures were subjected to a 2-h incubation with a primary antibody mixture: a polyclonal chicken anti-GFAP (a marker of differentiated astrocytes) (1:500 dilution, Abcam ab4674, United Kingdom) and polyclonal rabbit anti MAP2 (a marker of differentiated neurons) (1:500 dilution, Abcam 32454, United Kingdom). The cells were then washed three times with 0.1% Triton X-100 and 0.1% Tween 20 in PBS, followed by 2-h incubation in the secondary antibody mixture: goat anti-chicken Alexa 555 (1:800 dilution, Thermo Fisher Scientific, A-21437, United States) and chicken anti-Rabbit Alexa Fluor 647 (1:800 dilution, Thermo Fisher Scientific, A-21245, United States). The immunostained cultures were examined using a LSM 800 confocal laser-scanning microscope (Carl Zeiss, Germany).

### Cytotoxicity analysis *in vitro*

The viability of cells cultivated in the presence of scaffolds was estimated by the ratio of the number of dead cells stained with propidium iodide (5 mg/ml, Sigma-Aldrich, P4170, Germany) to the total number of cells stained with bisbenzimide (1 mg/ml, Invitrogen, H3570). The fluorescent dyes were added to the culture medium 30 min before viability was measured (Vedunova et al., 2015). For each stained culture, 10 fields of view were analyzed under an Axio Observer A1 (Zeiss, Germany) fluorescence microscope (10/0.2Ph1 objective). The analysis was performed in three independent experiments with five replicates in each. Cell viability was also assessed using a cytotoxicity score according to ISO 10993-5-2009 (https://www.iso.org/standard/36406.html).

### Calcium imaging

Functional Ca^2+^ activity in primary hippocampal cultures was assessed on day 14 of cultivation by a Ca^2+^ imaging technique using a Zeiss LSM 800 confocal laser-scanning microscope (Carl Zeiss, Germany) and an Oregon Green 488 BAPTA-1 AM (OGB-1) calcium sensor (0.4 mM, Thermo Fisher, United States). OGB-1 was excited at 488 nm and recorded in the range of 500–530 nm. Time series images of 512 × 512 pixels of 420 × 420-mm fields of view were recorded at 2 Hz. Detection and further analysis of Ca^2+^ oscillations were performed in the Astroscanner program (Vedunova et al., 2013; Mishchenko et al., 2019). The following parameters of spontaneous Ca^2+^ activity were analyzed: the percentage of working cells (the cells number with at least one recorded oscillation divided by the total cell number, %) and the duration (the time period from the beginning to the end of an oscillation, s) and frequency of Ca^2+^ oscillations (an average number of oscillations per min).

### Traumatic brain injury model and scaffold implantation *in vivo*

For biocompatibility studies *in vivo*, we used a model of traumatic brain injury (TBI). A more detailed description of the procedure is provided in our previous article (Novozhilova et al., 2020). Briefly, adult male C57BL/6 mice were subjected to open mechanical brain trauma by a weight-drop method (Feeney et al., 1981; Flierl et al., 2009; Zhang et al., 2014), with modifications. The animals were anesthetized with an intraperitoneal injection of Zoletil 100 (70 mg/kg, Virbac Sante Animale, France) and Хylanite (0.02 mg/kg, NITA-PHARM, Russia). Next, we performed a craniotomy with a fine drill (2-mm length and 2-mm diameter) in the right hemisphere near the central suture to the right of the lambda-bregma intersection, leaving the dura mater intact. An 80-cm plastic tube was placed vertically on the brain damage site. A blunt load weighing 4 g was dropped down the tube onto the abovementioned area. The wound was then sutured and treated with an antiseptic solution. After withdrawal from anesthesia, the animals were returned to their cages with postoperative care and *ad libitum* access to food and water.

Seven days later, the mice were anesthetized, and the operating field was prepared. The damaged brain tissue was carefully removed, and the scaffold was placed directly onto the injury site. The size of the implant was as close as possible to the injury volume. The wound was then sutured and treated with an antiseptic solution, and the mice were returned to their cages.

The animals were divided into the following groups:

1) Sham: the mice not subjected to surgical procedures;
2) TBI: traumatic brain injury without SC implantation;
3) TBI+SC: traumatic brain injury followed by implantation of SC devoid of neurotrophic factors;
4) TBI+SC+BDNF: TBI followed by implantation of SC loaded with the neurotrophic factor BDNF (100 ng/scaffold);
5) TBI+SC+GDNF: TBI followed by implantation of SC loaded with the neurotrophic factor GDNF (100 ng/scaffold).

### Neurological status determination

The dynamics of the functional state of the CNS was evaluated by using a scale for assessing the severity of neurological deficit with modifications for mice. The scale includes 30-s tests of motor activity, coordination of movements, reflexes, muscle tone, and ptosis and exophthalmos. A brief description of the performed tests is presented in Supplementary Information (Supplementary Table S1). Each test is scored two points for no reaction, 0 for good/normal reaction, and 1 for some disturbances. The values were summarized and interpreted as severe CNS damage (10–20 points), moderate damage (6–9 points) or light damage (1–5 points) (Prickaerts et al., 1999; Beni-Adani et al., 2001).

### Open field test

The general locomotor and orienting-exploratory activity of the experimental animals were tested in the Open Field Box (LE800S; Panlab Harvard Apparatus, Spain) in the early and late periods after SC implantation. The behavior of the mice was recorded for 5 min using a Sony SSC-G118 camera (Japan). The following reactions were assessed: vertical motor activity (the number of upright postures), emotional state (the number of grooming acts, defecation and urination) and the time spent in the center of the arena.

### Morris water maze

The Morris water maze test was conducted in a circular pool (90-cm diameter) filled with turbid warm water. A movable platform (10-cm diameter) was placed in a certain place of the pool 1–2 cm below the water surface. The animals were trained for 5 days. Each session consisted of three sessions of 60 s. Each mouse was placed on different sides of the pool to train them to find the platform by an external visual landmark. If the animal could not find the platform by the end of 60 s, it was placed on it. Long-term memory retention was assessed by testing the mice in a pool without a platform for 1 min. The delayed coefficient of retention (dCr) was calculated as the ratio between time spent in the area where the platform was previously located to the total time spent in the pool (Fox et al., 1998; Faden et al., 1999). The type of strategy the mice used to search for the platform was also recorded.

### Magnetic resonance imaging

To assess the dynamics of brain tissue changes at the scaffold site, magnetic resonance imaging (MRI) was applied using a high-field magnetic resonance tomograph Agilent Technologies DD2-400 9.4 T (400 MHz) with a volume coil M2M (Н^1^).

The animals were kept under general anesthesia in a fixed position inside the magnet tunnel for 40 min. The VnmrJ program was used to obtain and process data. T1- and T2-tomograms of layer-by-layer frontal brain sections weighted by proton density were performed using the multi gradient echo multi slice (MGEMS) pulse sequence with the following parameters: TR = 1000 ms, TE = 1.49 ms, six echoes, FOV 20 × 20 mm, matrix 256 × 256, slice thickness 1 mm, 15 slices, 17 min and 4 s scanning time. To obtain diffusion-weighted images, the spin echo multi slice (SEMS + diffusion) pulse sequence with the following parameters was used: TR = 1200 ms, TE = 2 ms, FOV 20 × 20 mm, matrix 256 × 256, slice thickness 1 mm, number of slices 15, scanning time 18 min.

### Morphological assessment

For histological studies, the brains were isolated and fixed in 10% formalin solution at room temperature for 2 days and then placed in 15% sucrose solution (24–48 h) followed by 30% sucrose solution (24–48 h). The samples were then transferred to a Leica CM1520 freezing sliding cryostat (Leica, Germany) and gradually filled with cryogel (Leica, Germany). The brain was cut into 15-µm coronal sections, which were placed on slides and dried in the air for 24 h. They were stained according to a standard hematoxylin-eosin method (PanReac AppliChem, Germany). Next, the slices were dehydrated in alcohol solutions of increasing concentrations, purified in xylene, and embedded in mounting medium (Thermo Fisher Scientific, United States). The samples were examined using a Zeiss Primo Star light microscope (Zeiss, Germany) with an integrated Axio CamMRc camera (Zeiss, Germany).

### Statistical analysis

Statistics was calculated in GraphPad Prism (V. 6.0). *In vitro* studies were performed in three independent experiments, each experimental and control groups included five cultures. The data on dynamics of the development of neuronal processes were collected from 10 fields of view of each culture and analysed using the Wilcoxon *t*-test. Cytotoxicity analysis was performed using one-way ANOVA followed by Dunnett’s multiple comparison test. The results of Ca^2+^ imaging were analysed using one-way ANOVA followed by Tukey post hoc test. *In vivo* studies were performed in two independent experiments; the number of mice for each group/time-point comprise 10–15 individual animals. The data were analysed using the Mann-Whitney test (for independent samples) and the Wilcoxon test (for dependent samples) and presented as “M [Q1; Q3],” where M—median, Q1—first quartile (quantile 0.25), and Q3—third quartile (quantile 0.75). Differences between groups were considered significant if the corresponding *p*-value was less than 0.05.

## Results

### Scaffold formation

**FIGURE 2:**
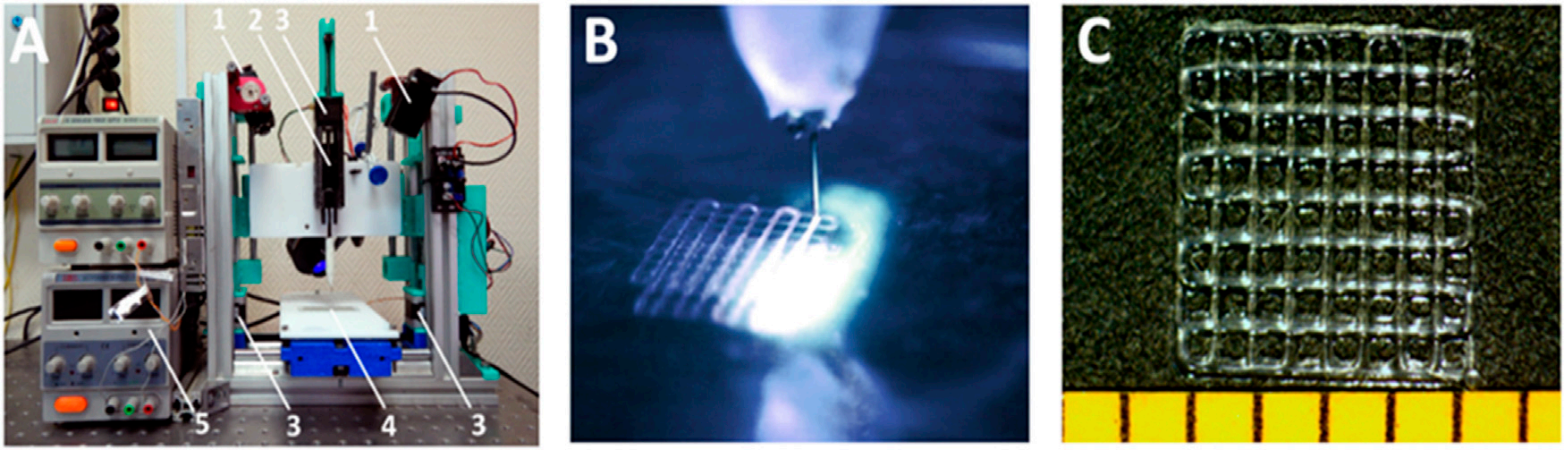
3D extrusion printing with simultaneous photocross-linking. **(A)** extrusion setup: 1) CW lasers; 2) extruder; 3) stepper motors; 4) sample compartment; 5) power supply. **(B)** photocuring of extruded hydrogel. **(C)** hydrogel structure on the substrate.

Scaffolds were fabricated from PCC on a base of HAGM with DS = 43%. 3D extrusion printer equipped with photocuring 450-nm lasers (Figure 2A). Figure 2B illustrates CCD image of printing process. Irradiation of the structure produced layer by layer can be observed. The printed samples (Figure 2C) represent five-layer gratings (4.3 × 4.3 mm) with a period of 540 µm. The cross-linked hydrogel had a Young’s modulus of 2.2 MPa. Relatively high SWR measured as 336% provides possibility to load scaffolds easily with any bioactive moiety soluble in water solutions.

The samples were divided into three groups. The first group (control scaffold, SC) was placed in distilled water and the second (SC + BDNF) and third parts (SC + GDNF) in aqueous solutions of rhBDNF and rhGDNF (30 ng/μl). Excess liquid was carefully removed. Thus, the concentration of growth factors in the scaffolds was ∼100 ng/scaffold. The soaked scaffolds indicated Young’s moduli decrease down to 0.6 ± 0.2 MPa, caused by decreased density of polymer lattice after swelling. More than 3-fold decrease of Young’s modulus makes mechanical properties slightly closer to those of brain tissue ranged as 1 kPa (Leipzig and Shoichet, 2009).

### Evaluation of cytotoxic and bioactive properties of the scaffold material *in vitro*

As the designed and fabricated 3D hyaluronic scaffolds were loaded with neurotrophic factors BDNF and GDNF, we aimed not only to evaluate the cytotoxic effects but also to assess the peculiarities of the formation and functional activity of neuron-glial networks on the background of the release of biologically active agents from scaffolds material. To achieve this goal, we analyzed the outgrowth dynamics of neuronal processes in the adjustment and distant areas of primary hippocampal cultures relative to the scaffold.

In the early stage of cultivation in the presence of scaffolds, neuronal processes formed and branched in the primary hippocampal cultures, which resulted in formation of numerous connections between cells. There were no significant differences in the number of neurites between the experimental groups in the adjacent or the distant areas of the cultures (*p* > 0.05) (Supplementary Table S2). During the first week of cultivation, the length of neuronal processes gradually increased in all the groups (Table 1). Notably, the inclusion of BDNF in the scaffold caused short-term stimulation of neurites development. On DIV 1 in the scaffold-adjacent area of the SC+BDNF group cultures, the growth in length of the neurites in both directions significantly exceeded the values of the sham group (toward the scaffold: Sham 15.11 ± 0.86 µm, SC+BDNF 23.34 ± 1.46 µm; away from the scaffold: Sham 16.12 ± 0.86 µm, SC+BDNF 21.85 ± 1.46 µm, *p* < 0.05). On day 3 of cultivation, the neurites in the adjacent and distant culture areas in the SC+BDNF group were still longer than in the sham group (toward the scaffold: Sham 26.90 ± 1.43 µm, SC+BDNF 37.26 ± 1.98 µm; away from the scaffold: Sham 23.93 ± 1.06 µm, SC+BDNF 30.52 ± 1.59, *p* < 0.05).

**TABLE 1:** Dynamics of the development of neuronal processes in the primary hippocampal cultures in the early stage of cultivation *in vitro.*

A: Adjacent area						
Experimental group	Length of neurites [µm]					
	DIV 1		DIV 3		DIV 7	
	Toward the scaffold	Away from the scaffold	Toward the scaffold	Away from the scaffold	Toward the scaffold	Away from the scaffold
Sham	15.11 ± 0.86	16.12 ± 0.86	26.90 ± 1.43	23.93 ± 1.06	50.33 ± 4.06	48.25 ± 3.62
SC	15.51 ± 0.84	14.18 ± 0.91	35.53 ± 1.77*	30.48 ± 1.86*	66.88 ± 5.80	60.32 ± 3.31
SC+BDNF	23.34 ± 1.46*	21.85 ± 1.46*	37.26 ± 1.98*	30.52 ± 1.59*	59.14 ± 4.01	57.72 ± 4.85
SC+GDNF	15.56 ± 0.78	15.16 ± 0.75	34.04 ± 1.99*	26.72 ± 1.04	54.65 ± 3.45	50.21 ± 3.74

B: Distant area						
Experimental group	Length of neurites, µm					
	DIV 1		DIV 3		DIV 7	
	Toward the scaffold	Away from the scaffold	Toward the scaffold	Away from the scaffold	Toward the scaffold	Away from the scaffold
Sham	15.44 ± 1.00	15.42 ± 1.03	24.42 ± 1.84	30.74 ± 2.41	54.28 ± 2.62	53.28 ± 4.15
SC	14.70 ± 0.92	13.38 ± 1.01	28.40 ± 1.73#	27.97 ± 1.77	55.81 ± 4.85	45.63 ± 3.48
SC+BDNF	15.12 ± 0.94#	13.86 ± 0.83	37.48 ± 3.12*	31.88 ± 4.10	44.73 ± 4.60	37.35 ± 4.42*
SC+GDNF	15.56 ± 0.78	13.91 ± 0.71	28.52 ± 1.66	32.46 ± 1.93#	55.51 ± 3.63	48.06 ± 5.10

The values are the mean ± standard error of the mean and represent three independent experiments with five replicates in each. Statistical significance was calculated by Wilcoxon *t*-test. * versus Sham, # versus the values from the adjacent area, *p* < 0.05.

Culturing primary hippocampal cultures with a control scaffold and a scaffold loaded with GDNF ensured active neuronal outgrowth on DIV 3 (toward the scaffold: SC 35.53 ± 1.77 µm, SC+GDNF 34.04 ± 1.99 µm, *p* < 0.05). By day 7 of cultivation, the length of neuronal processes was the same as the sham values (*p* > 0.05).

**FIGURE 3:**
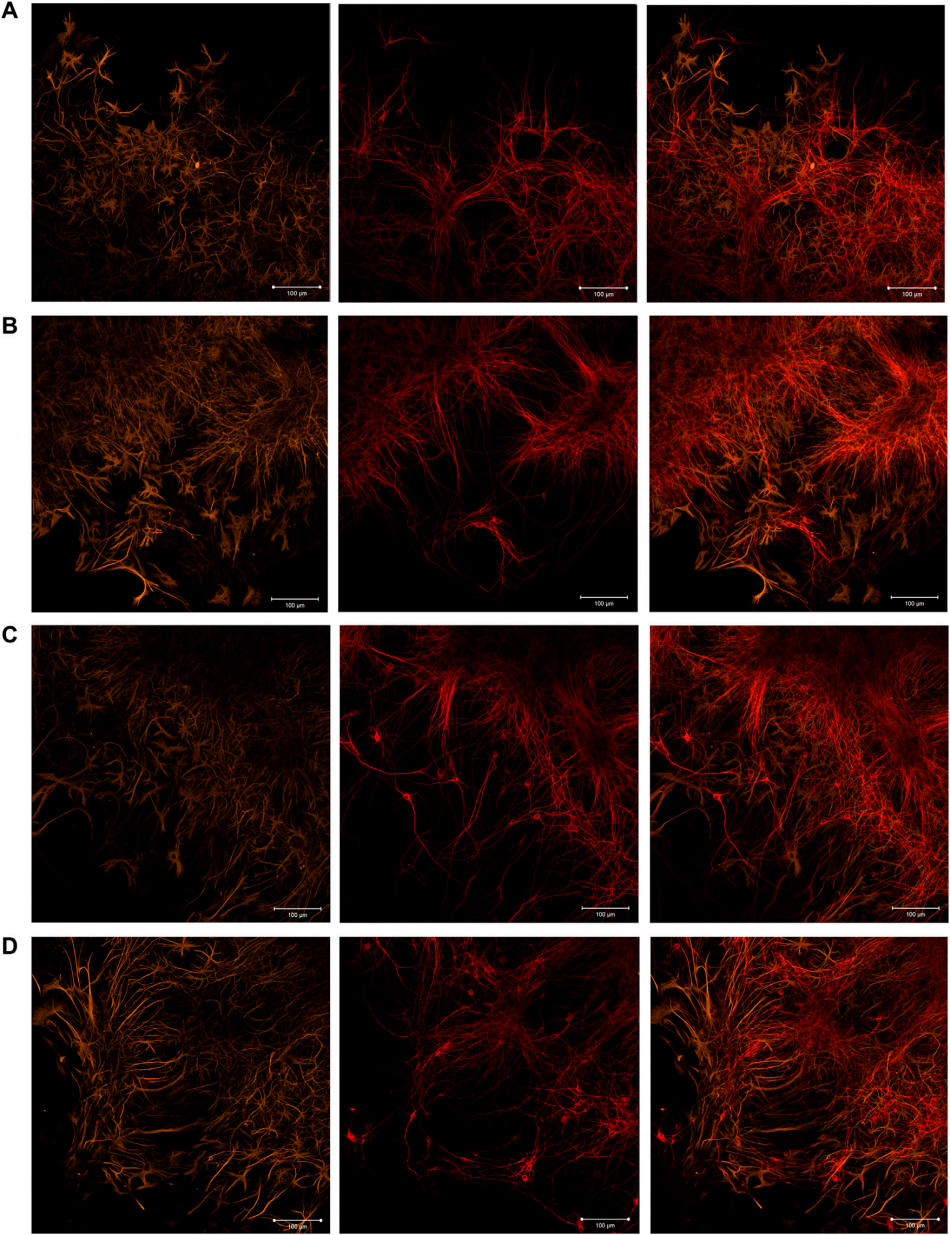
Immunocytochemical analysis of primary hippocampal cultures on day 7 of cultivation *in vitro*. Representative confocal images were obtained at the adjustment area of cultures relative to the scaffold. **(A)** Sham; **(B)** SC; **(C)** SC + BDNF; **(D)** SC + GDNF. Yellow: fluorescence of a marker of cytoskeleton protein of differentiated astrocytes (GFAP) (λ_ex_ 488 nm; λ_em_ 555–580 nm); Red: fluorescence of a marker of neuronal protein (MAP2) (λ_ex_ 594 nm; λ_em_ 650–665 nm); Merged: overlay of the fluorescence channels. Scale bars, 100 μm.

The immunocytochemical analysis performed on day 7 of cultivation revealed that the cellular content of primary hippocampal cultures culturing in the presence of scaffolds is represented by neurons and astrocytes in an approximate ratio of 1:2, which interact with each other by numerous connections and form the neuron-glial networks (Figure 3 and Supplementary Figure S1). The results are consistent with our previous studies and evidence that the morphology of primary hippocampal cultures is typical for this period of development *in vitro* (Shirokova et al., 2013).

Cell viability analysis on day 14 of cultivation revealed no pronounced toxicity of the scaffold material (Table 2). In the SC+BDNF and SC+GDNF groups, the number of viable cells of the primary hippocampal cultures did not differ from the sham values (Sham 91.81 ± 0.97, SC+BDNF 91.67 ± 1.08, SC+GDNF 88.69 ± 1.2, *p* > 0.05). Culturing with a control scaffold led to a decrease in cell viability in the culture and slight cytotoxicity compared to the Sham group (SC 86.57 ± 1.15, *p* < 0.05).

**TABLE 2:** Cytotoxicity analysis of scaffolds for primary hippocampal cultures on day 14 of development *in vitro*.

Experimental group	Number of viable cells [%]	Cytotoxicity score [points]
Sham	91.81 ± 0.97	0
SC	86.57 ± 1.15*	1
SC + BDNF	91.67 ± 1.08	0
SC + GDNF	88.69 ± 1.2	1

Cytotoxicity score according to the ISO 10993-5-2009 standard: 0 - non-toxic (0–10% of dead cells in culture), 1 – light (10–20% of dead cells in culture), 2 – average (20–30% of dead cells in culture), 3 – significant (>30% of dead cells in culture). The values are the mean ± standard error of the mean and represent three independent experiments with five replicates in each. Statistical significance was calculated by one-way ANOVA followed by Dunnett’s multiple comparison test. * - versus Sham, *p* < 0.05.

**FIGURE 4:**
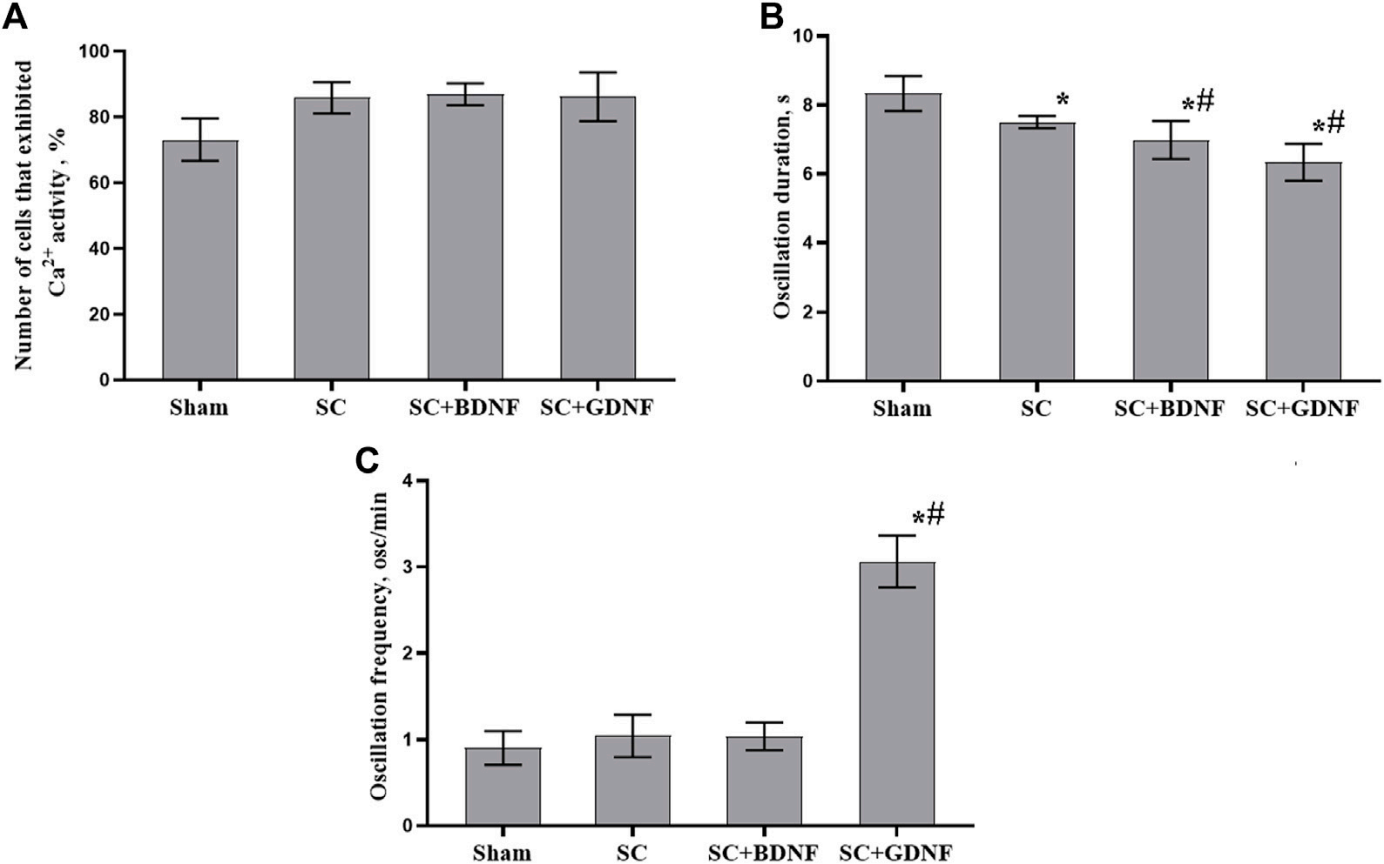
Main parameters of spontaneous calcium activity in primary hippocampal cultures on day 14 of development *in vitro*. **(A)** Proportion of cells exhibiting Ca^2+^ activity, **(B)** Duration of Ca^2+^ oscillations in seconds, **(C)** Number of Ca^2+^ oscillations per min. The values are the mean ± standard error of the mean and represent three independent experiments with five replicates in each. Statistical significance was calculated by one-way ANOVA and Tukey *post hoc* test. * - versus Sham, # - versus SC, *p* < 0.05.

No less significant aspect for understanding the reaction of nerve cells to the influence of scaffold material is the assessment of the functional neuron-glial network activity. Calcium imaging technique provides a powerful tool to characterize calcium dynamics in the cytoplasm of both neurons and astrocytes and assess the neural-glial network metabolic activity since it allows visualizing the architecture and mapping the activity of networks with cellular resolution (Savyuk et al., 2020; Mitroshina et al., 2021). The use of such approach allowed us to perform a more comprehensive assessment of cytotoxic effects of scaffold material and the risks of their possible side effects on brain cells. Assessment of the functional metabolic activity of neuron-glial networks revealed that the scaffold material modulates the spontaneous Ca^2+^ activity of primary hippocampal cultures (Supplementary Figure S2). While the number of functionally active cells remained unchanged, the duration of Ca^2+^ oscillations in the experimental groups was significantly modulated compared to that in the sham group (*p* < 0.05) (Figure 4). The most pronounced effects were in the SC + GDNF group: a 1.3-fold decrease in the duration of Ca^2+^ oscillations coupled with a 2.4-fold increase in the frequency of Ca^2+^ events (*p* < 0.05). These changes may indicate an enhancement of Ca^2+^ fluxes through the plasma membrane, which in turn activates the functional activity of neuron-glial networks.

The absence of pronounced cytotoxic effects, the active neuronal process outgrowth, and the presence of functionally active neuron-glial networks of primary hippocampal cultures culturing in the presence of scaffolds suggest that the fabricated constructs are highly biocompatible. This points to the feasibility of conducting *in vivo* studies to determine the effectiveness of their use as implants after TBI.

### Alterations in neurological status and behavioral reactions of mice after traumatic brain injury followed by scaffolds implantation

**FIGURE 5:**
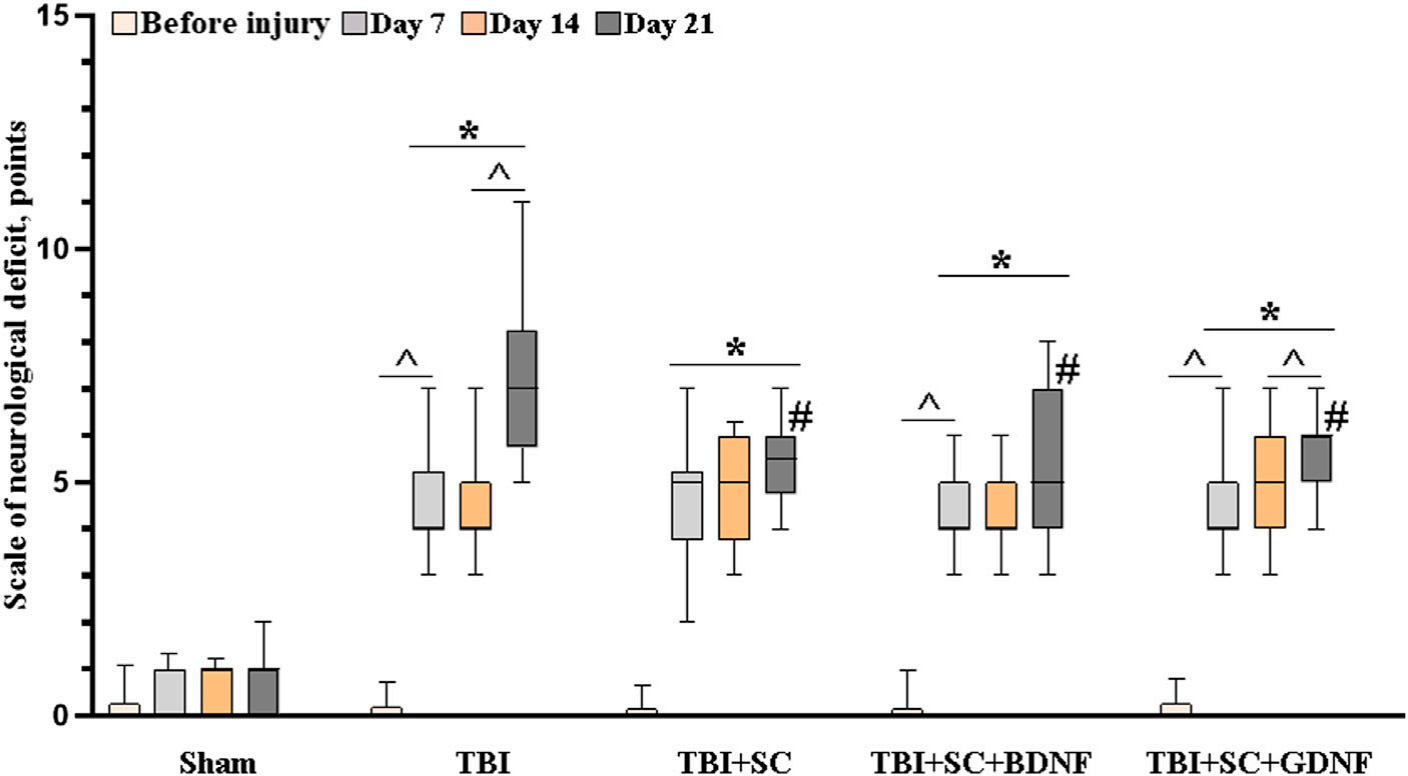
Neurological status assessment in mice after traumatic brain injury followed by scaffold implantation. Data presented as “M [Q1; Q3],” where M—median, Q1—first quartile (quantile 0.25), and Q3—third quartile (quantile 0.75) of two independent experiments; the number of mice for each group comprise at least 10 individual animals. Statistical significance was calculated by the Mann-Whitney test * - versus Sham, # - versus TBI, *p* < 0.05, and by the Wilcoxon test ^ - versus day 7, *p* < 0.05.

*In vivo* experiments showed that modeled TBI leads to the development of neurological deficits in mice (Figure 5).

In the third week of the post-traumatic period, neurological deficit in the TBI group reached 7 [5.75; 8.25], which corresponds to moderate CNS damage. Pronounced deficits in the performance of motor tasks, asymmetry of movements, proprioception of the body, and decreased responses to touching the vibrissae were observed. The use of scaffolds reduced the risk of development of severe neurological disorders. On day 21 after implantation, the values of neurological deficit in mice in the TBI+SC, TBI+SC+BDNF and TBI+SC+GDNF groups were 5.5 [4.75; 6], 5 [4; 7] and 5 [4; 6], respectively, which were significantly lower than the TBI values (*p* < 0.05). Nevertheless, minor CNS damage was observed, associated mainly with proprioceptive disorders. The mice from the TBI + SC group also showed impaired reflexes when tested for grasping the bar and the edge of any surface.

By the sixth month of the experiment, the neurological deficit in mice of all the groups decreased to values corresponding to light CNS damage (TBI 4 [3.5; 5.5], TBI + SC 4 [3; 4.5], TBI + SC + BDNF 4 [3; 4], TBI + SC + GDNF 3 [2; 4]). However, it remained higher than in the sham group (2 [0.5; 0.75] (*p* < 0.05).

Analysis of behavioral reactions in mice in the Open Field test on day 21 after scaffolds implantation revealed no pronounced alterations in orienting-exploratory activity and emotional status of animals compared to the sham group (Table 3). However, the mice in the TBI group tended to have lower motor activity in the arena center and lower vertical activity, which might have been associated with the parallel development of neurological deficits. The TBI+SC and TBI+SC+BDNF groups tended to have fewer upright postures, which suggests the possibility of emotional stress development.

**TABLE 3:** Parameters of behavioral reactions of mice in the Open Field test on day 21 after scaffold implantation.

A: Parameters of locomotor activity						
Experimental group	Number of squares passed in the arena		Time in the arena center [s]	Number of upright postures
	Periphery	Center		
Sham	93 [87.9; 107.5]	21 [14.5; 31]	42 [17.5; 50]	10 [6.5; 15.5]
TBI	83.5 [71.75; 102.3]	16 [8.25; 20]	28.5 [22; 37.5]	7 [4.25; 13.25]
TBI+SC	92.5 [82; 103.3]	22.5 [10.5; 38.75] #	40 [9; 52]	5 [2.75; 9.75]
TBI+SC+BDNF	87 [78; 102]	19 [15; 35] #	32 [24; 51]	6 [2; 11]
TBI+SC+GDNF	87 [78; 100]	23 [20; 35] #	44 [30; 57] #	8 [5; 16]

B: Emotional status characteristics						
Experimental group	Time of grooming [s]	Number of peeks in holes	Acts of urination	Acts of defecation
Sham	4 [0.5; 5.5]	40 [32.5; 48.5]	0 [0; 0.5]	0 [0; 1]
TBI	0.5 [0; 3]	47 [26.75; 51]	0 [0; 1]	0 [0; 0]
TBI+SC	0.5 [0; 8.5]	36.5 [28.5; 54.5]	0 [0; 0]	0 [0; 0]
TBI+SC+BDNF	1 [0; 4]	39 [32; 41]	0 [0; 0]	1 [0; 3]
TBI+SC+GDNF	0 [0; 6]	37 [30; 41]	0 [0; 0]	1 [0; 2]

Data presented as “M [Q1; Q3],” where M—median, Q1—first quartile (quantile 0.25), and Q3—third quartile (quantile 0.75) of two independent experiments; the number of mice for each group comprise at least 10 individual animals. Statistical significance was calculated by the Mann-Whitney test. * - versus Sham *p* < 0.05, # - versus TBI, *p* < 0.05.

### Features of spatial learning and working memory of mice after traumatic brain injury and scaffolds implantation

In addition to the development of neurological deficit, TBI is accompanied by mnestic and cognitive functions impairments. Therefore, spatial learning and memory in mice in the late post-traumatic period were analyzed using the Morris water maze test.

The mice retained their learning ability after the modeled TBI (Table 4). By the fifth training session, the time spent to reach the platform was decreased (first session 53 [21.5; 60] s, fifth session TBI 7.5 [5; 9] s, *p* < 0.05); the tactic for target search became directional in general and characterized by circular and radial movements. The mice implanted with the control scaffold demonstrated reduced learning ability. The time spent to reach the platform in the fifth training session in the TBI+SC group (46.5 [15.5; 58.25] s) was not significantly different from the first training session (60 [57.5; 60] s) and tended to be longer than the sham values (12 [6; 20.5] s) (*p* > 0.05). In the first training session, the mice in the TBI+SC+BDNF (60 [59.75; 60] s) and TBI+SC+GDNF (60 [59.75; 60] s) groups also spent more time searching for the platform compared to the sham values (44.5 [31.25; 50.5] s) (*p* < 0.05). However, the search time decreased by the fifth training session. At the same time, the mice in the TBI+SC+GDNF group chose the most favorable trajectory of movement, which allowed them to reach the target in the shortest time (5.5 [4; 6.25] s) (*p* < 0.05) (Table 4).

**TABLE 4:** Analysis of learning ability of mice in the Morris water maze after traumatic brain injury followed by scaffolds implantation.

Experimental group	Average time spent to reach the platform, s	
	*First training session*	*Fifth training session*
Sham	44.5[31.25; 50.5]	12 [6; 20.5]
TBI	53 [21.5; 60]	7.5 [5; 9] #
TBI+SC	60 [57.5; 60]	46.5 [15.5; 58.25]
TBI+SC+BDNF	60 [59.75; 60] *	25.5 [11.25; 51] #
TBI+SC+GDNF	60 [59.75; 60] *	5.5 [4; 6.25] *#

Data presented as “M [Q1; Q3]”, where M—median, Q1—first quartile (quantile 0.25), and Q3—third quartile (quantile 0.75) of two independent experiments; the number of mice for each group comprise at least 10 individual animals. Statistical significance was calculated by the Mann-Whitney test * - versus Sham, *P* < 0.05 and the Wilcoxon test # - versus first session, *P* < 0.05.

**FIGURE 6:**
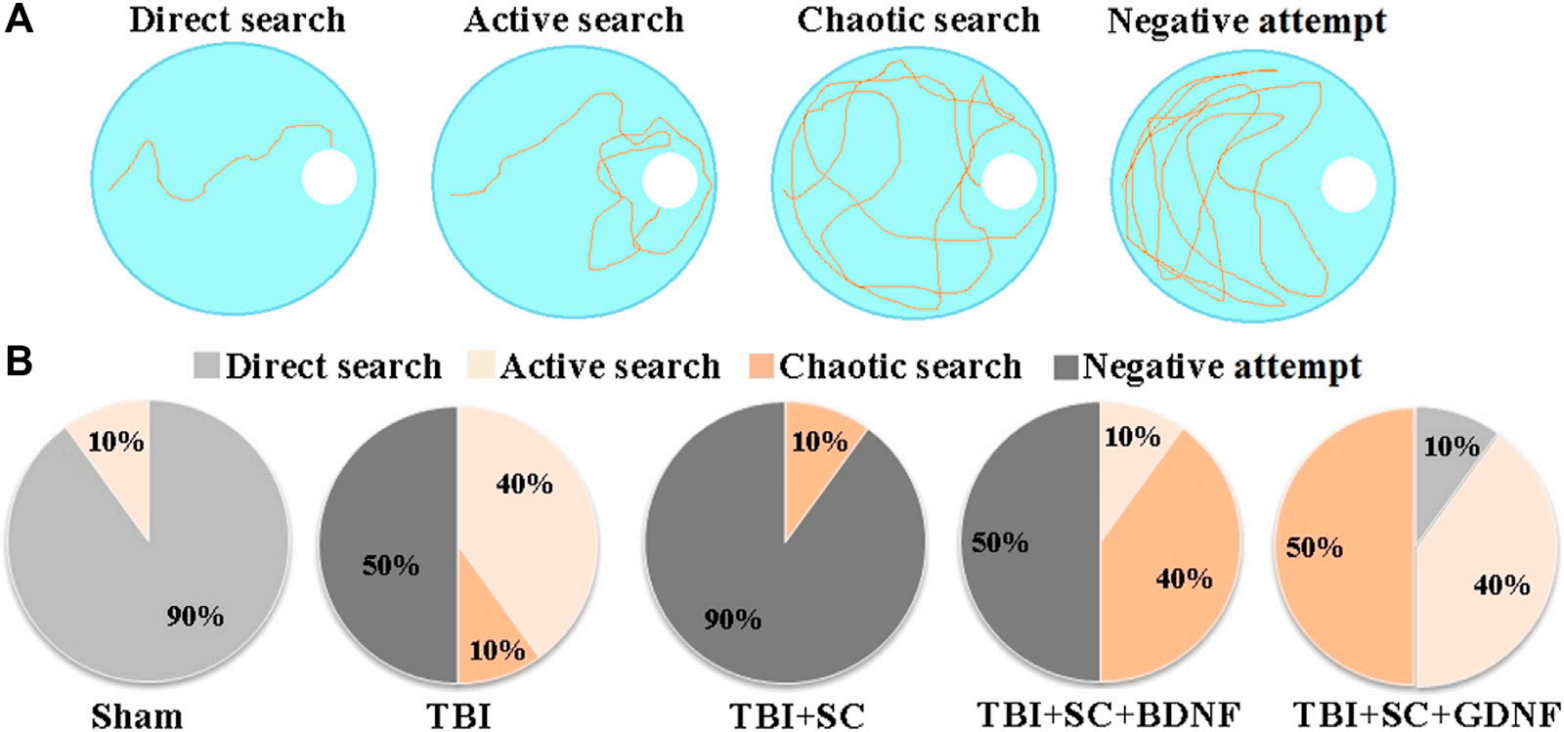
Behavioral traits of mice during long-term memory retention test in the Morris water maze. **(A)** Main target-searching strategies of mice in the Morris water maze; **(B)** Distribution of target-searching strategies in the experimental groups of mice.

Delayed testing in the Morris water maze (without the platform) showed that TBI impairs retention of long-term memory (Table 5 and Figure 6). The mice in the TBI group headed towards the platform immediately after the beginning of the test (5.25 [4.25; 5.87] s) but later changed their trajectory and spent more time in other pool sectors. Analysis of their platform search strategy revealed that in 50% of the cases the mice did not find the platform (Figure 6).

**TABLE 5:** Main parameters of long-term memory retention test of mice in the Morris water maze.

Experimental groups	Time spent to reach the platform [s]	Time spent in the zone where the platform used to be located [s]	The delayed coefficient of retention (dCr) [%]
Sham	2.55 [2.42; 2.9]	33.5 [26.75; 38.75]	48.66 [40.91; 56.41]
TBI	5.25 [4.25; 5.87]*	15.75 [13.5; 17.63]*	37.32 [33.41; 41.23]
TBI+SC	51 [44.25; 59.25]*#	13.75 [12.25; 16.38]*	18.77 [14.75; 22.79]*
TBI+SC+BDNF	6.5 [5.25; 8.5]*§	16.5 [15.25; 19.25]*	28.2 [26.55; 29.85]
TBI+SC+GDNF	16 [14.25; 19.25]*#§	20.65 [16.25; 24.08]	44.17 [44; 44.33]

Data presented as “M [Q1; Q3]”, where M—median, Q1—first quartile (quantile 0.25), and Q3—third quartile (quantile 0.75) of two independent experiments; the number of mice for each group comprise at least 10 individual animals. Statistical significance was calculated by the Mann-Whitney test * - versus Sham, # - versus TBI, § - versus TBI+SC, *p* < 0.05.

The animals in the TBI+SC group showed severe impairment of long-term memory and reduced learning ability. The dCr was 18.77 [14.75; 22.79]%, beyond the normal level but significantly lower than in the sham group (48.66 [40.91; 56.41]) (*p* < 0.05). In 90% of the cases, a negative attempt to find a target was observed (Figure 5). The mice spent the least amount of time in the pool sector where the platform was previously located (Table 5).

Mice in the TBI+SC+BDNF group headed towards the platform area in the beginning of the test (6.5 [5.25; 8.5] s) but later made chaotic movements in the pool. In 50% of the cases, a negative target search attempt was recorded. The dCr value was 28.2 [26.55; 29.85]% and tended to be lower than in the sham group.

The use of scaffolds loaded with GDNF reduced the severity of mnestic functions impairment after modeled TBI. The mice in the TBI+SC+GDNF group demonstrated active searching for the platform. These mice did not make negative attempts to find the target (Table 5). The dCr values and the length of time spent in the pool sector where the platform was previously located did not differ from the sham values (*p* > 0.05).

Thus, among the constructs studied, the application of scaffold impregnated with GDNF into the injury site has a more favorable impact on the maintaining of cognitive and mnestic functions in mice in the posttraumatic period.

### Dynamics of brain morphological changes in mice after traumatic brain injury followed by scaffolds transplantation to the injury site

**FIGURE 7:**
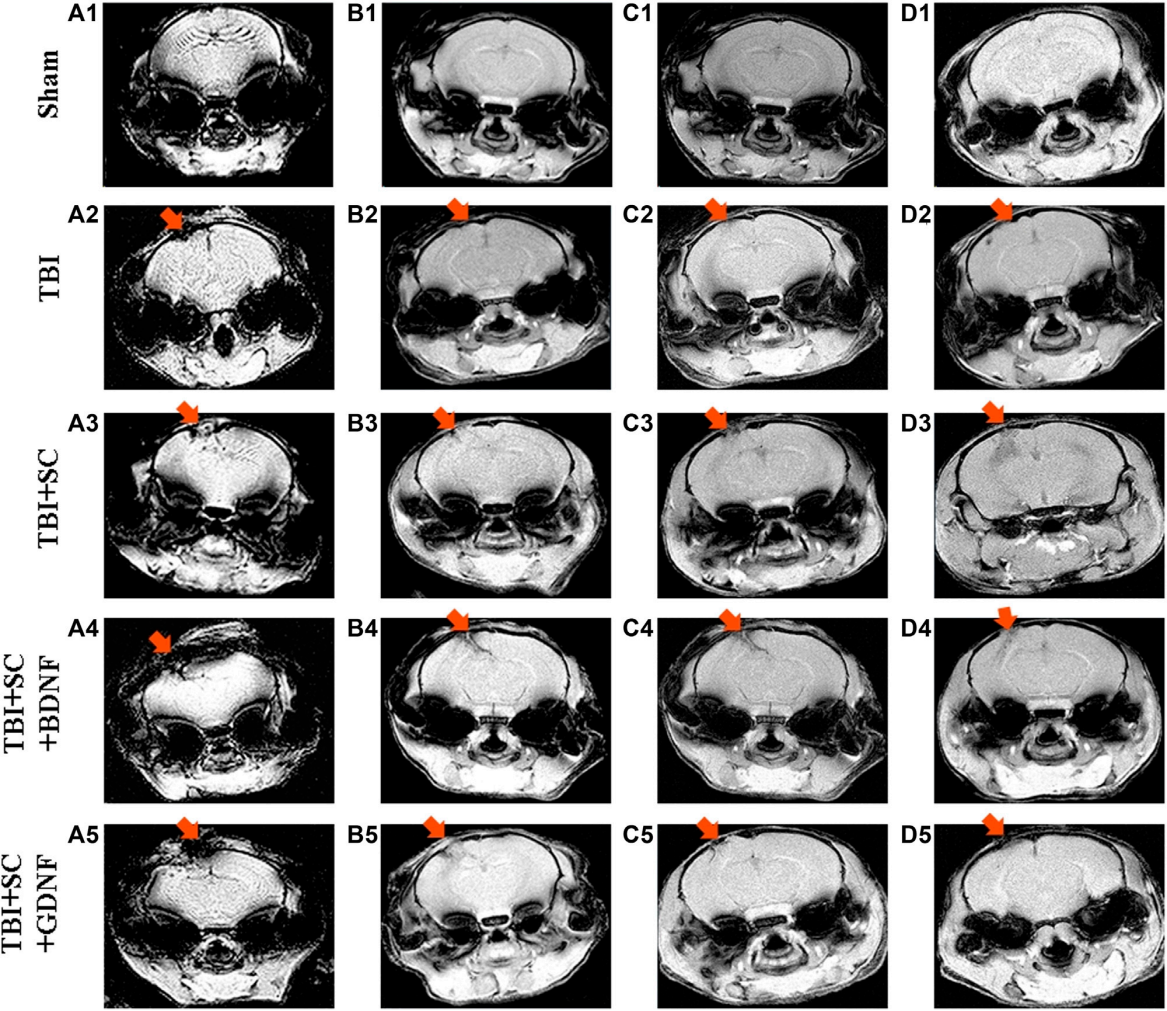
Representative MRI MGEMS images of mouse brains after modeled traumatic brain injury and scaffolds implantation. **A1-A5:** 7 days after implantation, **B1-B5:** 21 days after implantation; **C1-C5:** 2 months after implantation; **D1-D5:** 6 months after implantation.

MRI assessment of the dynamics revealed severe morphological impairments in the brain tissue of mice after TBI (Figure 7 and Supplementary Figure S3). MRI images obtained 2 weeks after TBI demonstrated indistinct contours of the sensorimotor cortex at the injury site (Figure 7 A2). By day 21, tissue shift and strong diffusion indicated the presence of necrotic masses and pronounced edema in the lesion (Figure 7 B2). A glial scar at the injury site was formed by the sixth month of observation (Figure 7 D2); the volume of tissue edema was 62.5[57; 68] mm^3^.

On day 21 after implantation of the control scaffold, MRI images showed active contact between the construct and the nerve tissue (Figure 7 B3). The scaffold then moved deep into the nerve tissue; SEMS_DWI showed a weak fluid inflow to the implantation site 2 months after implantation. After 6 months, the scaffold location did not change, but the surface areas of tissue expansion formed a commissure between them (Figure 7 D3). The volume of tissue edema did not differ from TBI values (67 [64; 70] mm^3^, *р* < 0.05, the Mann-Whitney test).

The positive dynamics was observed in animals with scaffolds loaded with neurotrophic factors. The TBI+SC+BDNF group showed the least noticeable signs of inflammatory reactions 7 days after implantation compared to the other experimental groups (Figure 7 A4-D4). By day 21 after implantation, MRI images demonstrated a significant decrease in the amount of fluid at the lesion site, and the two damaged brain areas converged at the implantation site (Figure 7 B4). Two months after implantation, the scaffold had pulled together the entire nerve tissue and filled the remaining damaged area (Figure 7 C4). After 6 months, the consequences of trauma and implantation were minimal: there were partial irregularities at the sensorimotor cortex surface and poorly visible contours of scaffold fragments in the deeper layers of nerve tissue (Figure 7 D4). The volume of tissue edema had a tendency to decrease related to the TBI group (45.5 [40; 51] mm^3^, *р* < 0.05, the Mann-Whitney test).

On day 21 after implantation of the scaffold loaded with GDNF, the MRI images revealed tissue destruction and development of diffuse inflow at the injury site, which indicated the presence of inflammatory reactions (Figure 7 A5-D5). However, nerve tissue growth and filling of voids in the injury site were noted in the TBI+SC+GDNF group 2 months after the modeled TBI (Figure 7 C5). Complete tissue fusion in the damaged area was evident 6 months after scaffold implantation (Figure 7 D5). There was weak diffusion and outgrowth on the right hemisphere surface, indicating regeneration activity. The volume of tissue edema was significantly decreased compared to the TBI values (40 [38; 42] mm^3^, *р* < 0.05, the Mann-Whitney test).

**FIGURE 8:**
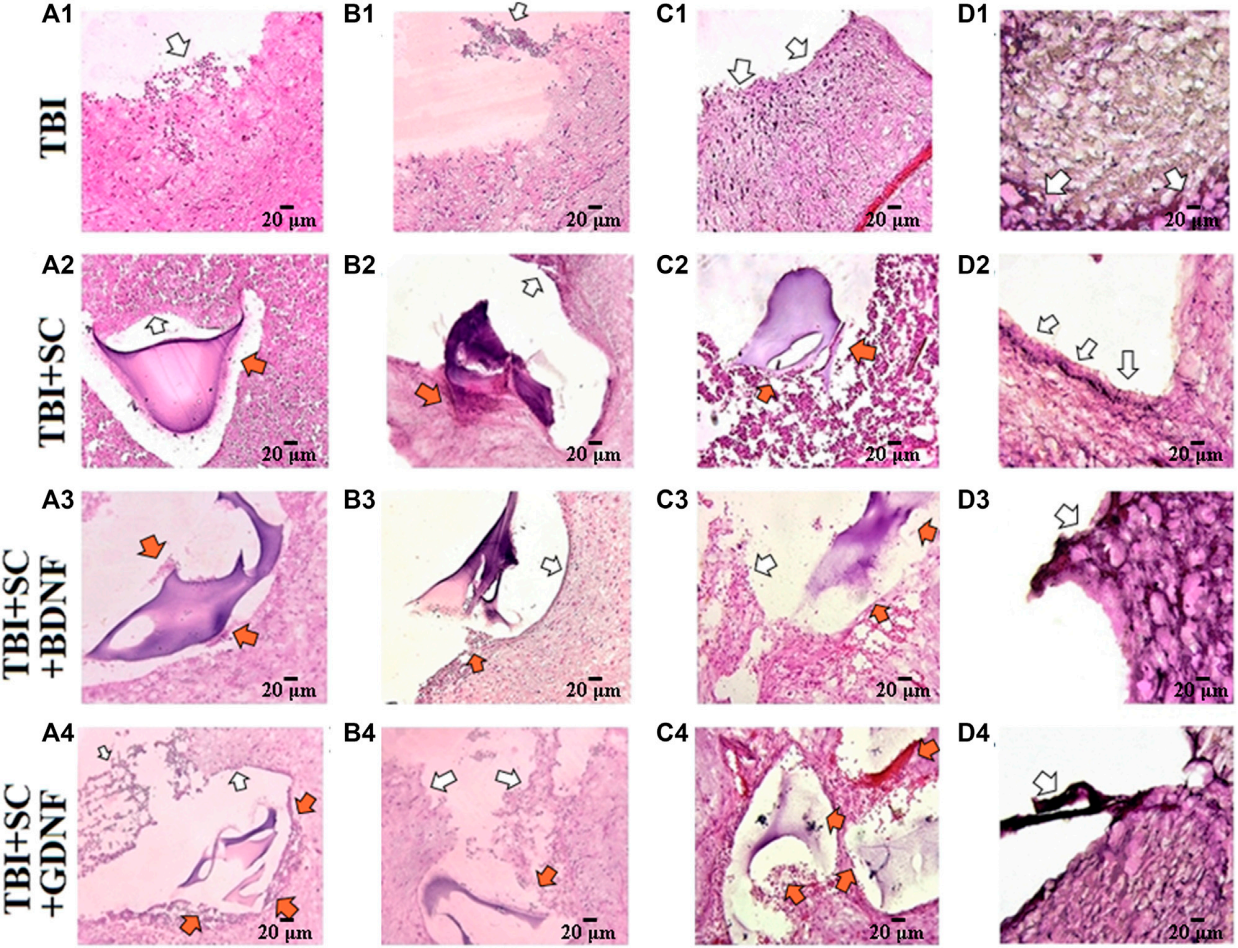
Representative histological samples of brain cortex of mice after traumatic brain injury and scaffolds implantation. Hematoxylin-eosin staining, magnification ×20. **A1-A4:** 7 days after implantation, **B1-B4:** 21 days after implantation; **C1-C4:** 2 months after implantation; **D1-D4:** 6 months after implantation. White arrows indicate the sites of nerve tissue expansion and signs of inflammation. Orange arrows indicate the sites of neuronal process outgrowth toward the scaffold Scale bars, 20 μm.

Histological analysis confirmed significant changes in the brain cortex morphology in the post-traumatic period (Figure 8). Two weeks after the TBI, there was complete loss of the brain tissue structure, massive edema, and extensive foci of necrosis and apoptotic elements of nerve cells in the histological preparations of the TBI group (Figure 8 A1). A focal, mild, lymphoid infiltration on the edge of the injury site was detected. Fourteen days after TBI, extensive foci of tissue expansion and its pre-necrotic changes were identified (Figure 8 B1). Three weeks after TBI, the brain tissue structure was completely disrupted (Figure 8 C1). Tissue lysis predominated. Single viable neurons were noted in 10 fields of view. Six months after TBI, extensive edema and tissue lysis led to the formation of an unstructured substance at the injury site (Figure 8 D1).

Weak positive dynamics were observed in the TBI+SC group (Figure 8 A2-D2). In the control scaffold group, there was nerve tissue expansion and slight hemorrhage from blood vessels 14 days after implantation (Figure 8 B2). The regenerative areas (approximately 2–3 small cellular agglomerates per 10 fields of view) were detected 3 weeks after implantation. Lysis of deeper layers of tissue predominated. Moreover, there was multiple tissue scarring and loss of the dense arrangement of cellular structures. Six months after implantation (Figure 8 D2), the tissue structure around the implant was characterized by strong cell adhesion; multiple accumulations of microglia and blood cells in 10 fields of view were noted. Scaffolds underwent structural changes and lost their mechanical properties. The residual fragments of the construct were located separately from the nerve tissue.

On day 7, the inflammatory reactions at the injury site in the TBI+SC+BDNF group were less pronounced after implantation than in the TBI group (Figure 8 A3). However, edema and foci of tissue expansion persisted throughout the experiment. Nevertheless, active processes of neuronal regeneration (approximately four groups of cells per 10 fields of view) and hypervolemia were detected on day 14 after scaffold implantation. A minor glial scar was detected near the implant. The development of tissue edema led to a decrease in the number of new neurons and an increase in the number of apoptotic and necrotic bodies by day 21 after scaffold implantation (Figure 8 B3). After 6 months (Figure 8 D3), the tissue structure became heterogeneous, and there was a decrease in the edema volume and in the sites of tissue expansion in both the cortex and the deeper layers of the brain. The walls of small blood vessels were substantially thickened, pointing to active regeneration processes.

Necrotic processes in the superficial cell layers and the areas of tissue expansion and edema in the deeper brain layers were observed on day 7 after implantation of the scaffold loaded with GDNF (Figure 8 A4). Nevertheless, new neurons (approximately three groups of cells per 10 fields of view) and remarkable hypervolemia were detected at the implantation site. The edema of the surface layers of the brain cortex and foci of tissue expansion were still present on day 14 after surgery. There was remarkable hypervolemia and active regeneration (approximately eight groups of cells per 10 fields of view), which became less intense by day 21 (about three to four groups of cells) (Figure 8 B3). Six months after implantation, the brain morphology resembled that in the TBI+SC+BDNF group (Figure 8 D3). However, more intensive neuronal process outgrowth was noted in the TBI+SC+GDNF group. The tissue edema was decreased, and perivascular space limen was moderate.

## Discussion

Fabrication of scaffolds with specific structure intended for transplantation after surgery or brain injuries of various origins is one of the strategic directions in biomedical materials science. Brain tissue has a complex heterogeneous structure (*e.g*., different cell types, biomolecules, blood vessels, fibrous proteins), and its functions are crucial for normal body functions. Therefore, development of constructs for neurotransplantation has special requirements in terms of chemical composition, biological activity, cytotoxicity level, and structure (they should mimic the replaced tissue, *i.e*., have a certain porous structure, mechanical strength, *etc*.) (Mahumane et al., 2018; Oliveira et al., 2018).

In this study, we used 3D extrusion printing to fabricate scaffolds for damaged tissue repair (Ning and Chen, 2017). This technique allows rapid fabrication of 3D structures from a wide range of biomaterials (Später et al., 2020). The scaffold can be designed easily by using computer software, and parameters of extrusion are corrected depending on the viscosity of PCC. To date, it is the most convenient technique for regenerative medicine applications because it is simpler and deposition is faster than in other methods, such as two-photon polymerization (Kufelt et al., 2014) or photolithography (Bobula et al., 2017).

To exclude the development of cytotoxic effects and maximize approximation to the native brain tissue composition, hyaluronic acid formed the basis of our scaffolds design. HA, a natural polymer, is the main component of the brain extracellular matrix, performs a wide range of functions, and contributes to maintenance and homeostasis of the CNS (Senkov et al., 2014; Perkins et al., 2017; Song and Dityatev, 2018; Jensen et al., 2020). As a natural component of brain tissue, biodegradation of hyaluronic acid follows physiological patterns, minimizing the possibility of formation of undesirable metabolic products. The high hydrophilicity of HA also means that scaffolds formed on its basis have good adhesive properties for cells providing a favorable microenvironment for them (Abatangelo et al., 2020). Moreover, HA can be modified chemically by simple methods that enable cross-linking of polymer chains to highly tunable scaffolds (Burdick and Prestwich, 2011; Xu et al., 2012; Highley et al., 2016; Choi et al., 2019; Ahmadian et al., 2020; Jensen et al., 2020; Spearman et al., 2020). It opens up the possibility of improving the physicochemical characteristics of scaffolds and controlling their rate of biodegradation. In the present work, HA was subjected to methacrylation to stabilize its mechanical properties. To modify HA by moieties containing double bonds, we conjugated HA with GMA by the chemical reaction of glycidyl groups with carboxylic and hydroxylic groups of HA. The synthesized biocompatible HA derivative (HAGM) can undergo photoinduced cross-linking. The spatial-temporal degree of hydrogel cross-linking during photo exposure depends on the illumination dose and DS.

*In vitro* studies have shown that scaffolds based on hyaluronic acid glycidyl methacrylate do not have a pronounced cytotoxic effect on nerve cells. Cultivation of primary neuronal cultures with control scaffolds did not significantly affect the rate of formation and branching of neuronal processes or the development of intercellular contacts, which allowed formation of functionally active neuron-glial networks. Implantation of control scaffolds after the modeled TBI contributed to a decrease in the risk of developing severe neurological deficit, substantial changes in orienting-exploratory activity, and changes in the emotional status of mice. Nevertheless, mice with an implanted control scaffold showed reduced learning ability and pronounced disturbances in long-term memory retention. The MRI images and histological studies showed active contacts of nervous tissue with the construct and formation of adhesions between the areas of nervous tissue expansion. However, tissue edema persisted for 6 months. Besides, there were multiple accumulations of microglia and blood, indicating the presence of inflammatory processes in the area of implantation, which could be one of the causes of the impairment of mnestic and cognitive functions in mice.

Following the primary traumatic injury associated with damage to the integrity of nerve and glial cells and brain blood vessels, the activation of biochemical cascades leads to secondary injuries. These include the development of excitotoxicity, oxidative stress, morpho-functional disorders in the mitochondrial apparatus due to the development of hypoxia/ischemic processes, changes in the permeability of the blood-brain barrier, neuroinflammation progression, and cytokine damage (Corps et al., 2015; Sevost’yanov et al., 2018; Ladak et al., 2019; Lazaridis et al., 2019). These molecular and cellular mechanisms can lead to the development of cytotoxic or vasogenic cerebral edema and impaired regulation, whereby the volume of intracranial contents increases due to vasodilation or water accumulation (Stocchetti and Maas, 2014). The development of these processes not only slows down nervous tissue regeneration but also leads to the death of previously undamaged cells.

Consequently, besides the need to maintain brain tissue structure, there is a need to include additional biologically active substances in the scaffold composition. These substances should provide the transplantation area with a favorable microenvironment to reduce the risk of developing secondary injuries after TBI. In this work, the neurotrophic factors BDNF and GDNF served as biologically active agents. These regulatory proteins are considered by many research groups as promising therapeutic agents for a wide range of CNS pathologies, including hypoxic-ischemic processes accompanying TBI (Benarroch, 2015; Wurzelmann et al., 2017; Zhao et al., 2017; Walker and Xu, 2018; Cintrón-Colón et al., 2020). Numerous experimental studies have shown that exogenous or endogenous stimulation of the production of the neurotrophic factors BDNF and GDNF has a pronounced neuroprotective effect that contributes to the preservation of cell viability, including cells not included in neuron-glial networks. They are also key participants in adaptations that allow maintenance of functional neural network activity under stress (Duarte et al., 2012; Mitroshina Е et al., 2018; Mitroshina Е et al., 2019; Lonser et al., 2020; Gustafsson et al., 2021). Besides, BDNF and GDNF are actively involved in the formation of neuronal processes and their outgrowth, maintenance of the structural and functional organization of the synaptic apparatus, and regulation of synaptic plasticity (Ibáñez and Andressoo, 2017; Leal et al., 2017; Kowiański et al., 2018; Mishchenko et al., 2019). Together, these can potentially provide the developed scaffolds with increased regenerative potential. In recent experimental studies, it has been observed that the loading the neurotrophic factors BDNF and GDNF into scaffolds of various composition enhances peripheral nerve regeneration in spinal cord injury (Tom et al., 2018; Hassannejad et al., 2019; Tajdaran et al., 2019; Ji et al., 2020; Cacialli, 2021), improve the survival and proliferation of transplanted neural cells (Nakaji-Hirabayashi et al., 2009; Wang et al., 2011) and provide the positive dynamics in brain tissue recovery in experimental stroke (Moshayedi et al., 2016; Cook et al., 2017).

We have shown in our *in vitro* studies that in addition to the absence of pronounced cytotoxic effects of BDNF and the active formation of primary neuronal cultures co-cultured with the scaffold, loading BDNF into the construct promoted the stimulation of neuronal process outgrowth on the first day of cultures development. Besides active participation in the neurogenesis of different stages of development (Huang and Reichardt, 2001; Vilar and Mira, 2016; Numakawa et al., 2018), BDNF can also control short- and long-lasting synaptic interactions and participates in maintaining neuron survival even in the temporary absence of connections between cells (Itami et al., 2003; Park and Poo, 2013; Kowiański et al., 2018; Colucci-D'Amato et al., 2020). Our recent studies also shows that chronic stimulation of BDNF signaling system contributes to the formation of more complex functionally active neural networks during the development of primary hippocampal cultures with a high level of synaptic transmission efficiency (Mishchenko et al., 2019). It can be assumed that the gradual release of the neurotrophic factor BDNF from the scaffold stimulated the development of neuronal outgrowth, primarily those in close proximity to the 3D construct.

On the other hand, the effect of GDNF was mainly aimed at increasing the functional activity of neuron-glial networks of primary hippocampal cultures at later stages of cultivation (DIV 14). GDNF does not directly bind to its receptor and implements such key functions as proliferation and survival of various populations of nerve cells as well as neuroprotection through the formation of an active complex with its receptors—GDNF/GFRα/Ret (Jing et al., 1996; Treanor et al., 1996; Airaksinen and Saarma, 2002; Ibáñez and Andressoo, 2017). However, a wide range of studies of the last decade provided a RET-independent GDNF signaling through GFRα1 and neural cell adhesion molecule NCAM (Ibáñez et al., 2020), which regulates different cellular processes, including synapse formation (Ledda et al., 2007; Irala et al., 2016), neurite outgrowth (Cao et al., 2008; Nielsen et al., 2009; Irala et al., 2016), axonal guidance and dendrite branching (Ledda et al., 2007; Irala et al., 2016; Bonafina et al., 2019). Due to the peculiarities of the metabolic cascades, the development of the effects of GDNF gradual release from the scaffold manifested in the modulation of synaptic transmission and activation of spontaneous calcium activity of primary hippocampal cultures. The effect was characterized by a significant increase in the frequency of Ca^2+^ oscillations, while the number of working cells was maintained at the sham values. The observations are most likely related to the ability of GDNF to increase the permeability of high-voltage Ca^2+^ channels which can lead to the enhancement of Ca^2+^ fluxes through the plasma membrane and eventually changes the excitability of nerve cells and activates the functional activity of neuron-glial networks (Doxakis et al., 2000; Wang et al., 2001; Wang et al., 2003).

Thus, despite the multifaceted actions of the neurotrophic factors on the formation and functioning of brain neuron-glial networks, the inclusion of both BDNF and GDNF in the scaffold composition can potentially increase the efficacy of using such constructs as neurotransplants after TBI.

*In vivo* studies have demonstrated that implantation of scaffolds with BDNF contributes to decreasing the neurological deficit of mice in the posttraumatic period. Histological analysis and MRI images showed the less pronounced inflammatory reactions in the injury site, the appearance of new neurons, thickening of the walls of small blood vessels and hypervolemia, pointing to active regeneration processes. The use of the BDNF-loaded scaffolds allows converging of the nerve tissue adjacent to the implantation site. However, the edema persisted throughout the experiment tended to suppress the intensity of the regenerative effects of the scaffolds with BDNF that characterized by the inhibition of the emergence of new nerve cells and the presence of apoptotic and necrotic processes in the implantation site. This was apparently one of the causes of some impairments in mnestic and cognitive functions in mice from the “TBI+BDNF” group. Taking into account the positive dynamics in nerve tissue regeneration, the main strategy for improving the efficacy of the BDNF-loaded scaffolds should be focused on the additional antiedematous therapy.

Scaffolds loaded with GDNF showed more favorable regenerative potential. According to MRI images and histological preparations, 6 months after implantation, there was a decrease in the edema volume and complete fusion of nerve tissue in the injury site and hypervolemia, preserving an appearance of new nerve cells and active processes of formation of intercellular connections and intensive neuronal process outgrowth. The ability of GDNF to stimulate the functional activity of cells (shown here *in vitro*) and regenerative processes had a positive effect on the physiological state of the mice. In addition to the absence of severe neurological deficit and significant changes in motor and orienting-exploratory activity, the mice preserved the ability to learn and retained long-term memory.

In spite of the favorable regenerative potential of scaffolds loaded with neurotrophic factors, they still reveal limitations in terms of mimicking the cellular matrix of brain tissue. In particular, to provide satisfied cell distribution within the volume of the scaffold, the cells should be already introduced in hydrogel during the fabrication of the matrix (Chen et al., 2020; Savelyev et al., 2021). Furthermore, it is challenging to implant the scaffold in the place of the defect in a minimally invasive way. To address these drawbacks in our future works, we plan to introduce cell-laden PCC into the body by injection through the needle, followed by cell-friendly photocuring to form the cross-linked hydrogel structure *in situ*. Another limitation of this study is that we did not examine scaffolds loaded with both neurotrophic factors (BDNF + GDNF). Taking into account a putative antagonistic action of neurotrophic factors BDNF and GDNF in their combined application (Vedunova et al., 2014; Mishchenko et al., 2018), it is necessary to find an optimal balance in concentrations of neurotrophic factors in the scaffolds composition to decrease the risk of side effects and achieve the acceleration of regenerative processes and functional recovery of nerve tissue in the posttraumatic period. That constitutes the goal of our upcoming research.

## Conclusion

In summary, we have shown in experimental studies *in vitro* and *in vivo* that the scaffolds we designed and fabricated based on hyaluronic acid glycidyl methacrylate by 3D extrusion printing are biocompatible with nervous system cells and could be useful for improving the development of strategies for morphological and functional nerve tissue restoration after TBI. The most favorable regenerative potential was found for scaffolds loaded with the neurotrophic factor GDNF. The positive effects we identified require further exploration. The exciting area for upcoming research is a search for the optimal/effective concentration and the optimal rate of neurotrophic factor release during scaffold biodegradation in the brain tissue *in vivo.*

## Data Availability

The raw data required to reproduce these findings are available on request.
