# Supplementary figures and images for: 3D-printed hyaluronic acid hydrogel scaffolds impregnated with neurotrophic factors (BDNF, GDNF) for post-traumatic brain tissue reconstruction
Source: Front Bioeng Biotechnol. 2022 Aug 25;10:895406. doi: 10.3389/fbioe.2022.895406 (PMC9453866; doi:10.3389/fbioe.2022.895406)

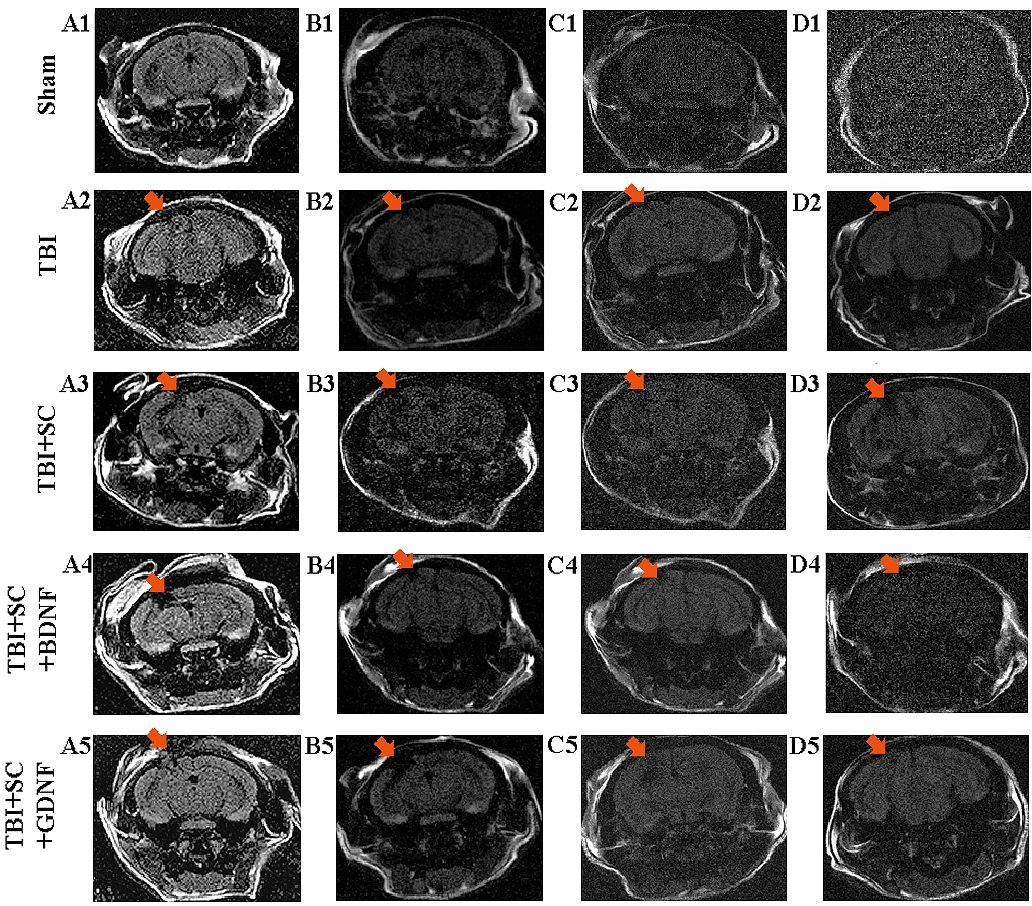

Supplement: Supplementary file 1 [file Image3.TIF]

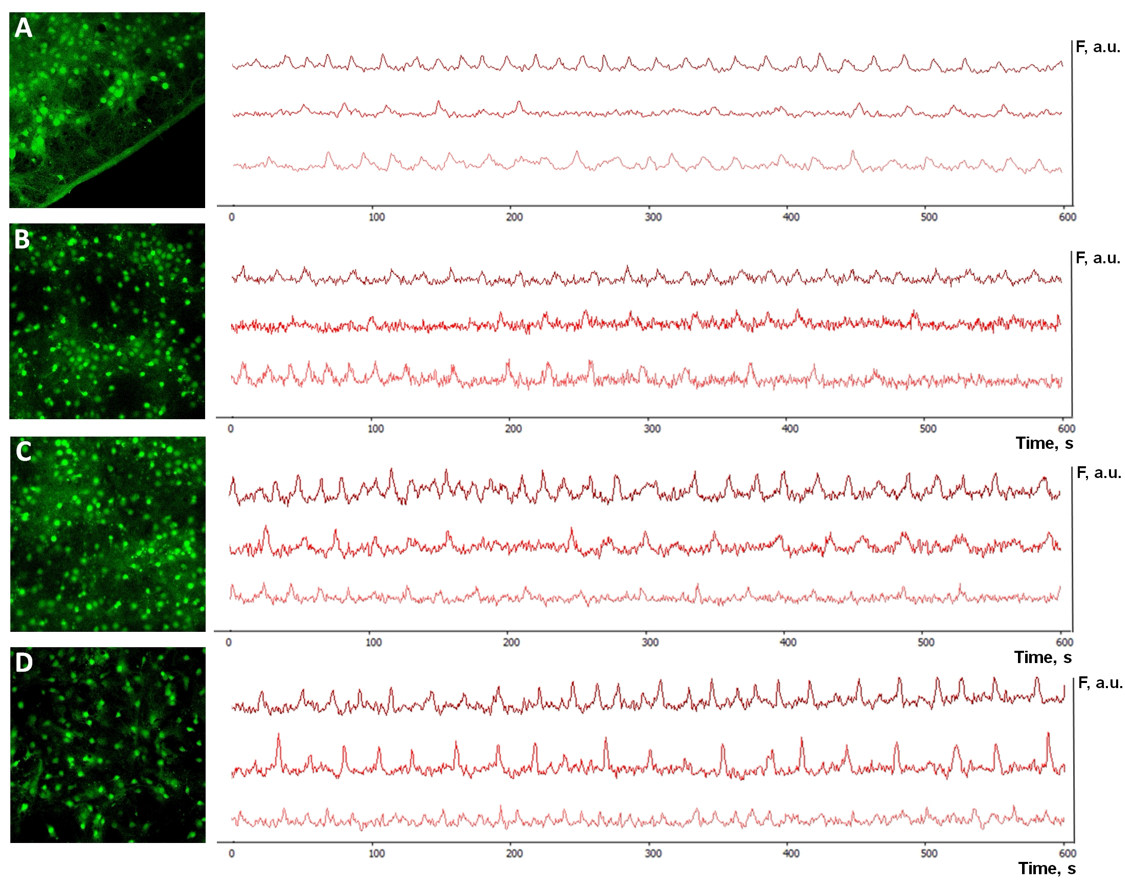

Supplement: Supplementary file 2 [file Image2.TIF]

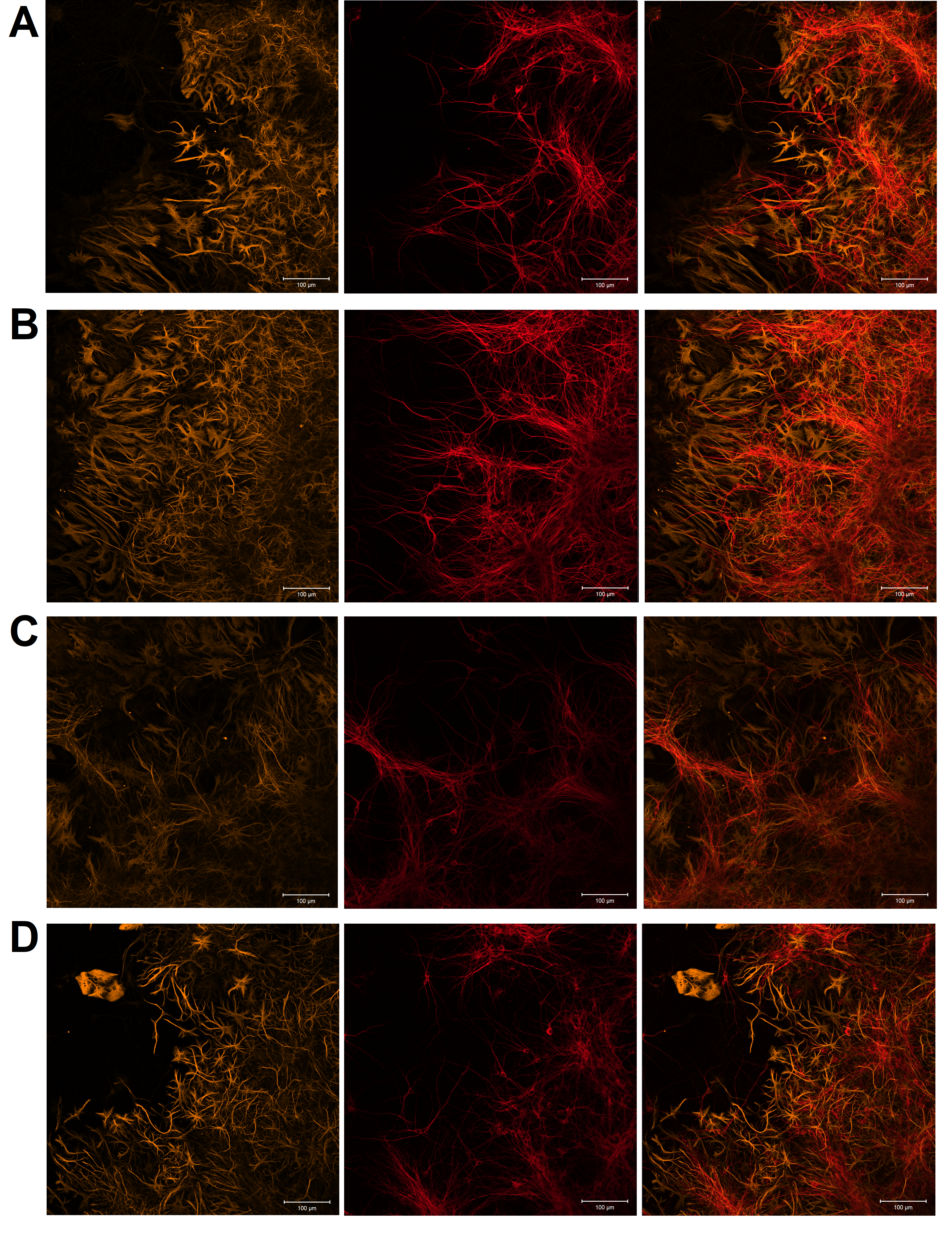

Supplement: Supplementary file 3 [file Image1.TIF]
