# Supplementary material for: 3D-printed hyaluronic acid hydrogel scaffolds impregnated with neurotrophic factors (BDNF, GDNF) for post-traumatic brain tissue reconstruction
Source: Front Bioeng Biotechnol. 2022 Aug 25;10:895406. doi: 10.3389/fbioe.2022.895406 (PMC9453866; doi:10.3389/fbioe.2022.895406)
Supplement: Supplementary file 4 [file DataSheet1.docx]

Supplementary Material

**SI Table 1.** Scale for assessing the severity of neurological deficit in mice

| **Test** | **Description** | **Score** |
| --- | --- | --- |
|  |  |  |
| Forelimb grasping reflex test | The animal is guided by the forepaws on the edge of a horizontal surface | 0 - holding with two paws;  1 – holding with alternating paws;  2 – lack of stretching the paws |
| Stretching the forelimbs to the vibrissae | The animal is lowered 5 cm above a horizontal surface not reaching the vibrissae | 0 – stretching to the surface with two paws;  1 – stretching to the surface with one paw or alternating paws;  2 – lack of stretching to the surface |
| Stretching the forelimbs reaching the vibrissae | The animal is lowered above a horizontal surface reaching the vibrissae | 0 – stretching to the surface with two paws;  1 – stretching to the surface with one paw or alternating paws;  2 – lack of stretching to the surface |
| Exiting the arena center | The animal is placed in the center of an “Open field” box for 30 seconds | 0 – leaving the arena center in 30 seconds;  1 – the animal took its forepaws out of the arena center and then came back;  2 – the animal did not leave the arena center |
| Trajectory of movement | The animal is placed in an “Open field” box to analyze its ability to maintain directional movements | 0 – multidirectional trajectory;  1 – the prevalence of circular movements with trajectory changes;  2 - circular movements |
| Ptosis | Low upper eyelid position in relation to the eyeball due to its prolapse | 0 – lack of ptosis;  1 – ptosis of one eye;  2 - ptosis of both eyes |
| Exophthalmos | Eyeball protrusion and displacement | 0 – lack of exophthalmos;  1 – exophthalmos of one eye;  2 - exophthalmos of both eyes |
| Beam-walking test | The animal is placed on a beam of 15 mm wide above a horizontal surface not reaching it. The animal is held on the beam by the forepaws only | 0 – confident balancing on the beam;  1 – the animal tried to grasp, but failed to climb the beam;  2 – the animal falls on a horizontal surface |
| Crossing the hind limbs | The animal is placed high above a horizontal surface | 0 – the animal is calm or only moves its hind limbs;  1 – the animal tried to pull up one paw;  2 – the animal crossed the hind limbs |
| Startle reflex test | The animal is pricked in the pad of the hind limbs with a sharp object (needle) | 0 – strong defensive reaction;  1 – weak defensive reaction;  2 – no defensive reaction |

**SI Table 2.** Dynamics of the development of neuronal processes in the primary hippocampal cultures in the early stage of co-cultivation with scaffolds *in vitro*

*A: Adjacent area*

| **Experimental group** | **Number of neuronal processes** | | | | | |
| --- | --- | --- | --- | --- | --- | --- |
|  | **DIV 1** | | **DIV 3** | | **DIV 7** | |
|  | Toward the scaffold | Away from the scaffold | Toward the scaffold | Away from the scaffold | Toward the scaffold | Away from the scaffold |
| Sham | 22.50±8.50 | 25.00±9.00 | 12.33±3.18 | 12.33±3.93 | 14.33±3.18 | 14.33±3.39 |
| SC | 27.50±11.50 | 18.00±5.99 | 24.00±11.50 | 17.33±6.33 | 18.33±1,46 | 17.67±1.33 |
| SC+BDNF | 24.50±7.50 | 14.00±2.00 | 25.00±8.39 | 20.33±5.93 | 14.67±0,33 | 13.67±0,67 |
| SC+GDNF | 23.00±2.00 | 20.00±4.00 | 24.67±9.67 | 20.00±5.69 | 16.00±2,65 | 13.00±2,00 |

*B: Distant area*

| **Experimental group** | **Number of neuronal processes** | | | | | |
| --- | --- | --- | --- | --- | --- | --- |
|  | **DIV 1** | | **DIV 3** | | **DIV 7** | |
|  | Toward the scaffold | Away from the scaffold | Toward the scaffold | Away from the scaffold | Toward the scaffold | Away from the scaffold |
| Sham | 17.00±5.99 | 19.00±7.00 | 12.67±6.94 | 12.67±5.78 | 17.00±3.61 | 14.67±4.63 |
| SC | 14.00±2.00 | 14.50±1.50 | 18.00±7.37 | 19.00±7.21 | 12.67±1.76 | 14.00±2.65 |
| SC+BDNF | 22.00±2.04 | 18.00±2.00 | 15.00±3.51 | 11.67±2.03 | 13.00±2.08 | 12.33±3.18 |
| SC+GDNF | 16.50±1.50 | 18.05±2.00 | 17.33±2.40 | 16.00±5.03 | 13.00±3.79 | 13.33±4.33 |

The values are the mean ± standard error of the mean and represent three independent experiments with five replicates in each. Statistical significance was calculated by Wilcoxon T-test. No statistical differences between groups (all p values > 0.05).


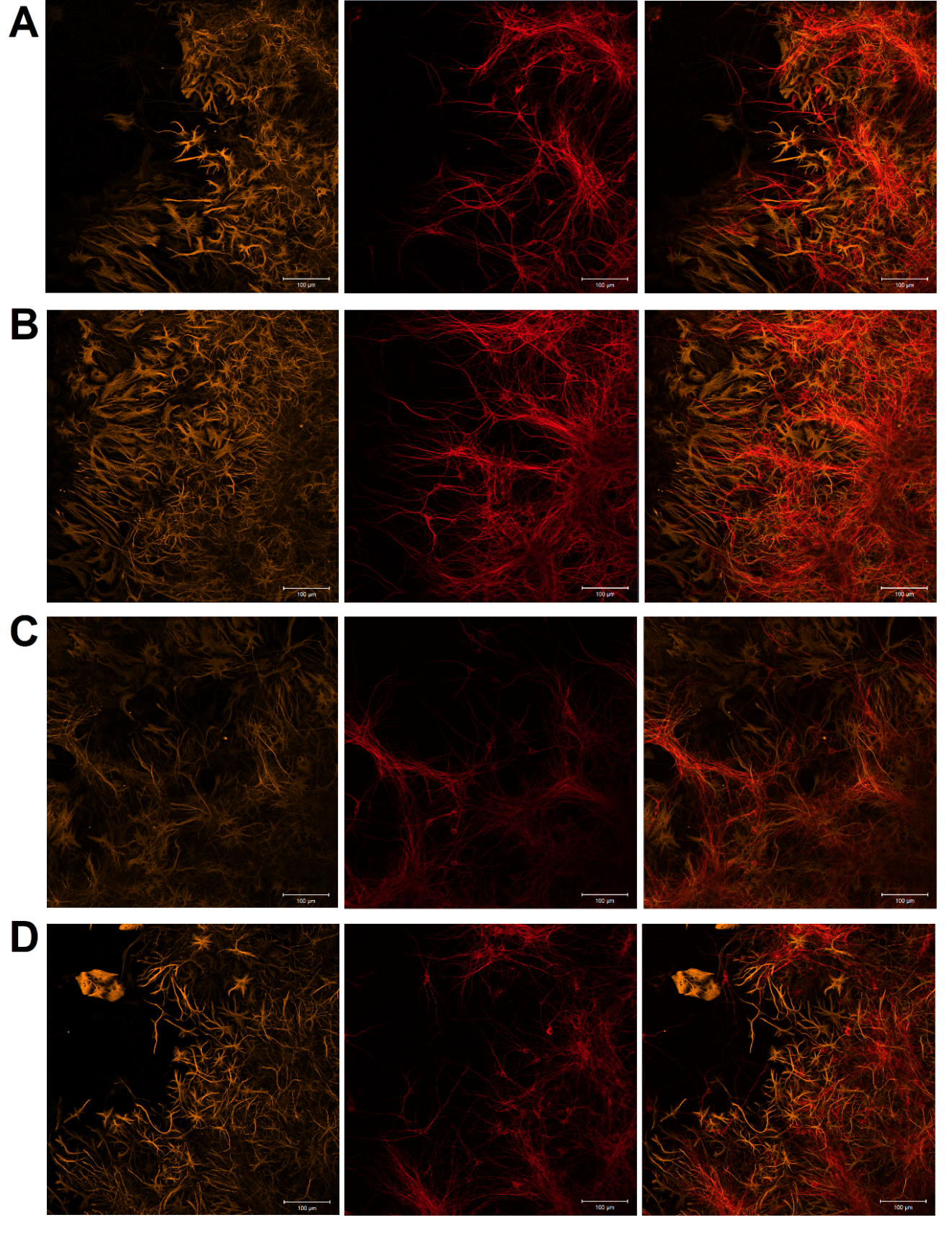


**SI Figure 1.** Immunocytochemical analysis of primary hippocampal cultures on day 7 of cultivation *in vitro*. Representative confocal images were obtained at the distant area of cultures relative to the scaffold. A: Sham; B: SC; C: SC + BDNF; D: SC + GDNF. Yellow: fluorescence of a marker of cytoskeleton protein of differentiated astrocytes (GFAP) (λ_ex_ 488 nm; λ_em_ 555-580 nm); Red: fluorescence of a marker of neuronal protein (MAP2) (λ_ex_ 594 nm; λ_em_ 650-665 nm); Merged: overlay of the fluorescence channels. Scale bars, 100 μm.


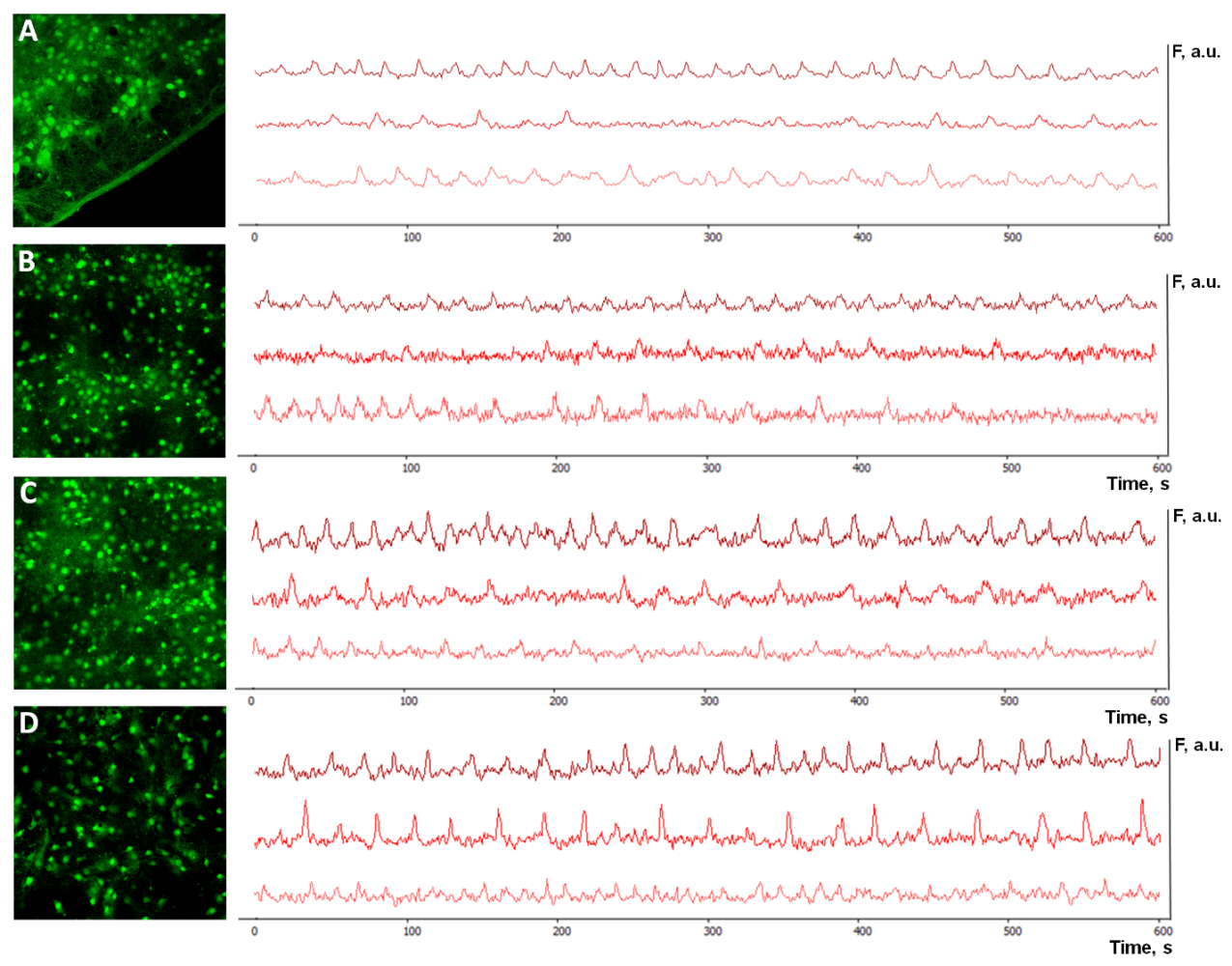


**SI Figure 2.** Representative recordings of spontaneous Ca^2+^ activity of primary hippocampal cells on day 14 of development *in vitro*. А: Sham, B: SC, C: SC+BDNF, D: SC+GDNF

**
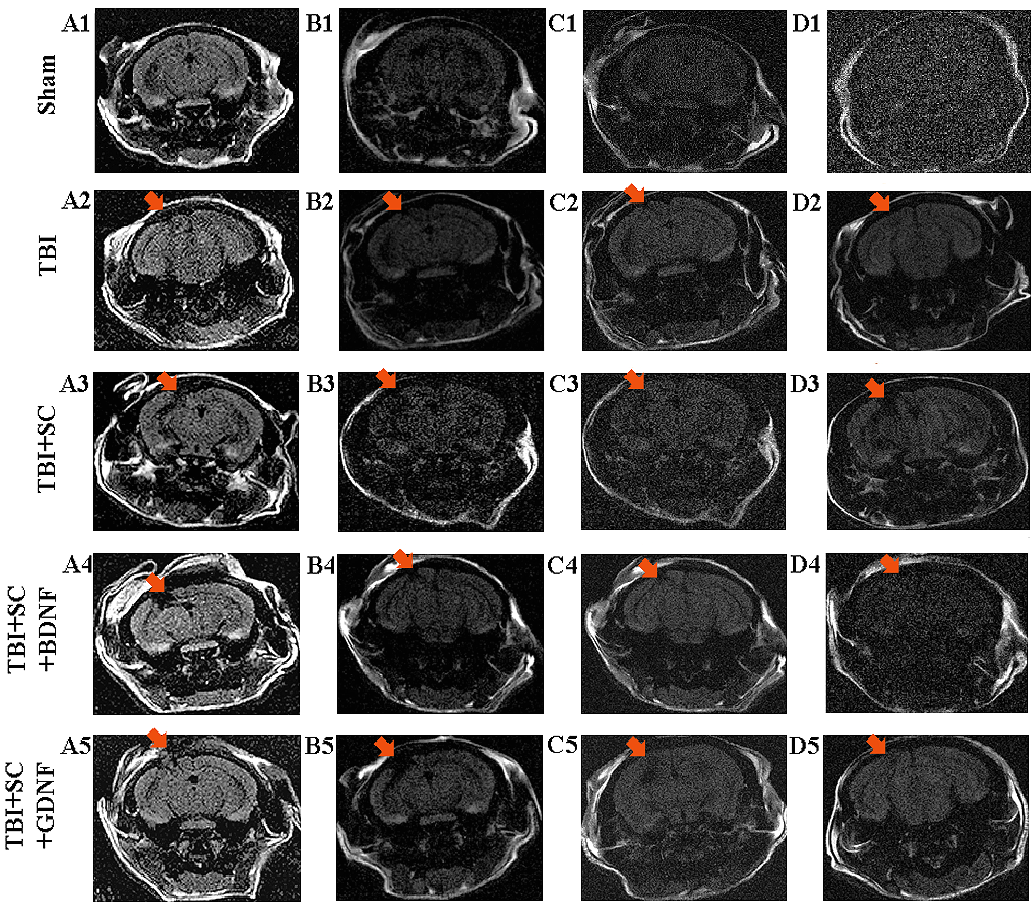
SI Figure 3.** Representative MRI SEMS+diffusion images of mouse brains after modeled traumatic brain injury and scaffolds implantation. A1-A5: 7 days after implantation, B1-B5: 21 days after implantation; C1-C5: 2 months after implantation; D1-D5: 6 months after implantation.
